# Targeting LDHA Palmitoylation Sensitizes GBM to Chemoradiotherapy via Suppressing Palmitic Acid Induced Glycolytic Reprogramming

**DOI:** 10.1002/advs.77536

**Published:** 2026-09-28

**Authors:** Zijie Gao, Rongrong Zhao, Zheyuan Chen, Qingtong Wang, Huanyu Yang, Boyan Li, Yanhua Qi, Shulin Zhao, Jixing Zhao, Jiawei Qiu, Ziwen Pan, Zhe Han, Ruochuan Li, Huize Xia, Weijie Tang, Xing Guo, Ping Zhang, Hongyu Zhao, Xu Qian, Gang Li, Hao Xue

**Affiliations:** ^1^ Shandong Key Laboratory of Brain Health and Function Remodeling Department of Neurosurgery Qilu Hospital of Shandong University Jinan Shandong China; ^2^ Institute of Brain and Brain‐Inspired Science Shandong University Jinan Shandong China; ^3^ Glioma Comprehensive Diagnosis and Treatment Center Qilu Hospital of Shandong University Jinan Shandong China; ^4^ Department of Neurosurgery Provincial Hospital Affiliated to Shandong First Medical University Jinan Shandong China; ^5^ Institute For Brain Tumors State Key Laboratory Cultivation Base of Biomarkers for Cancer Precision Prevention and Treatment Collaborative Innovation Center for Cancer Personalized Medicine Nanjing Medical University Nanjing Jiangsu China; ^6^ Department of Nutrition and Food Hygiene Center for Global Health School of Public Health Nanjing Medical University Nanjing Jiangsu China

**Keywords:** cancer research, chemistry, glioma, glycolysis, mesenchymal stem cell, palmitic acid, palmitoylation, reprogramming, stem cell, tumor microenvironment

## Abstract

Glioblastoma (GBM) is a lethal malignancy characterized by therapeutic resistance and recurrence, which are driven by dynamic cellular metabolic adaptation within the tumor microenvironment. However, the precise molecular metabolic mechanisms remain poorly understood. Here, we found that palmitic acid (PA) accumulates in hypoxic pseudopalisading regions of GBM and drives the formation of mesenchymal (MES) GBM and the hypoxia myeloid‐derived macrophages (MDM) subpopulation, establishing an interactive tumor‐promoting niche that fuels GBM recurrence. Maechanistically, PA induces ZDHHC12‐mediated palmitoylation of lactate dehydrogenase A (LDHA) at cysteine 163. This enhances LDHA enzymatic activity, promoting glycolytic reprogramming that reinforces the MES phenotype and confers resistance to chemoradiotherapy. Concurrently, palmitoylation facilitates the exosomal sorting of LDHA, which is internalized by MDMs, inducing their polarization into an immunosuppressive, glycolytic hypoxia‐MDM subset, which cooperatively shapes an immunosuppressive pseudopalisading niche with MES glioma stem cells to promote GBM recurrence. Critically, we identified the small molecule Rutin as a potent inhibitor of LDHA‐Cys163 palmitoylation, thereby re‐sensitizing GBM to chemoradiotherapy. This study defines a metabolic circuit in recurrent GBM driven by crosstalk between PA‐activated MES‐GBM cells and hypoxia‐MDMs. The small molecule Rutin disrupts this circuit, re‐sensitizing GBM to chemoradiotherapy and offering a promising strategy to overcome post‐treatment recurrence.

## Graphical abstract

Mesenchymal GSCs preferentially uptake palmitic acid, thereby promoting ZDHHC12‐mediated LDHA palmitoylation. This modification enhances LDHA enzymatic activity, drives glycolytic reprogramming, and facilitates exosomal LDHA‐mediated TAM regulation. Pharmacological targeting of the LDHA Cys163 palmitoylation site by Rutin markedly suppresses LDHA palmitoylation and sensitizes MES GSCs to chemoradiotherapy.

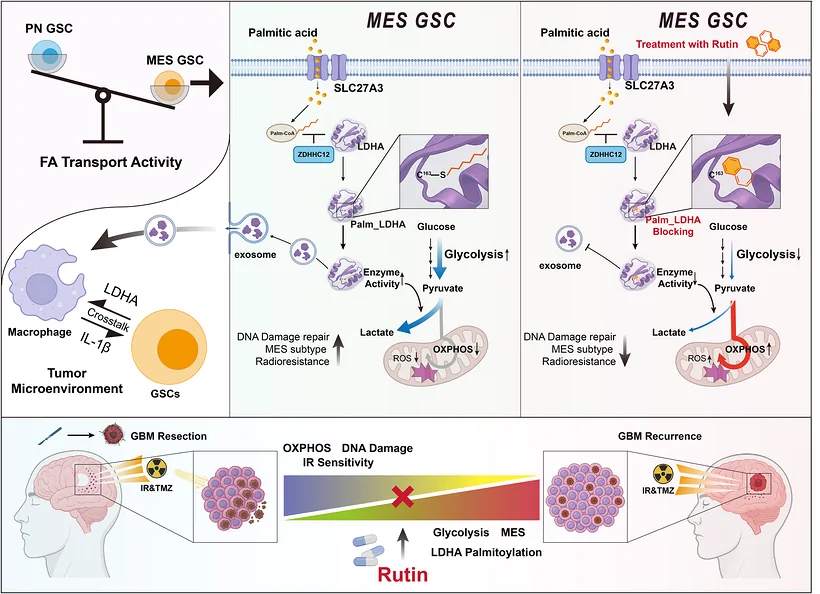

## 1 Introduction

Glioblastoma multiforme (GBM) represents the most aggressive primary intracranial malignancy, distinguished by near‐universal recurrence following multimodal therapy [1, 2, 3]. This therapeutic recalcitrance arises from profound intratumoral heterogeneity, wherein molecularly distinct cellular subpopulations exhibit differential susceptibility to conventional chemoradiotherapy [4, 5, 6]. Among them, the mesenchymal (MES) subtype, defined by high malignancy and aggressiveness, exhibits particularly pronounced resistance, thereby undermining therapeutic efficacy and compromising patient survival [7, 8]. Critically, a continuously sustained metabolic symbiosis between MES tumor cells and the immunosuppressive microenvironment collectively fosters the development of adaptive resistance mechanisms [9, 10, 11]. Thus, elucidating the niche‐specific metabolic drivers that coordinate cross‐compartment crosstalk to sustain therapeutic resistance is critical for developing targeted interventions.

Within this ecological landscape, hypoxic pseudopalisading necrosis emerges as a pathognomonic feature and independent predictor of poor prognosis [12, 13, 14]. These pathophysiological niches impose extreme metabolic stress on both malignant glioma stem cells (GSCs) and infiltrating stromal populations, activating parallel adaptation pathways including glycolytic rewiring, lipid metabolic reprogramming, and immunosuppressive signaling [15, 16, 17]. The precise mechanisms by which spatially localized metabolites within these microenvironments coordinate functional remodeling of malignant cells and immune cell populations to promote therapeutic tolerance remain incompletely defined. Elucidating this metabolic crosstalk is critical for developing effective microenvironment‐targeted therapeutic interventions.

Tumor‐associated myeloid cells (TAMs), the predominant immune infiltrates in GBM, encompass both resident microglia and infiltrating monocyte‐derived macrophages (MDMs) [18]. With tumor progression or recurrence, the proportion of MDMs progressively surpasses that of proinflammatory microglia, shifting the myeloid landscape toward a more immunosuppressive phenotype [19, 20, 21]. Critically, the GBM‐characteristic hypoxic microenvironment recruits MDMs and induces phenotypic reprogramming mediating therapeutic resistance [22, 23, 24]. Despite conserved hypoxic‐responsive MDM subsets in primary solid tumors, whether hypoxia‐driven MDM phenotypes dynamically evolve hierarchy during therapy, and how tumor metabolic fitness orchestrates the formation of these subsets require further investigation.

In this study, we found that palmitic acid (PA) accumulates in hypoxic pseudopalisading regions of GBM and drives the formation of MES GSCs and the hypoxia‐induced MDM subpopulation, establishing an interactive tumor‐promoting niche that fuels GBM recurrence. Mechanistically, PA drives ZDHHC12‐mediated palmitoylation of lactate dehydrogenase A (LDHA) at cysteine residue 163. This modification enhances LDHA enzymatic activity, thereby promoting glycolytic reprogramming, reinforcing the MES phenotype of GSCs, and conferring resistance to chemoradiotherapy. Concurrently, palmitoylation facilitates exosomal sorting of LDHA. The exosomal LDHA is subsequently internalized by MDMs, inducing an immunosuppressive glycolytic hypoxia‐MDM subset. This subpopulation cooperatively shapes an immunosuppressive pseudopalisading niche with MES‐GSCs to promote GBM recurrence. Critically, structure‐based virtual screening identified Rutin as a potent inhibitor of LDHA‐Cys163 palmitoylation. By disrupting this signaling axis, Rutin re‐sensitized GBM to chemoradiotherapy, and suppressed tumor progression. This work elucidated the potential tissue adaptation mechanism of intratumoral dynamic interactions between MES‐GBM cells and hypoxia‐MDM in recurrent GBM and identified the therapeutic potential of LDHA‐Cys163 palmitoylation to reduce therapy resistance in GBM.

## 2 Results

### 2.1 Hypoxic Pseudopalisading Necrosis Region Acquires Palmitic Acid‐Driven Pro‐tumor Recurrence Traits

Pseudopalisading necrosis regions in glioblastoma (GBM) are critically associated with patient prognosis and tumor recurrence [13, 25]. To identify metabolic pathways specifically upregulated in hypoxic necrotic zones, we performed integrated single‐cell RNA sequencing (scRNA‐seq), spatial transcriptomics (ST), and spatial metabolomics (SM) analyses on surgically resected tumor core and infiltrative margin specimens from six IDH‐wildtype GBM patients, comprising three primary and three recurrent cases (Figure 1A). Through unbiased single‐cell clustering coupled with canonical lineage marker expression profiling, we delineated transcriptionally distinct cell populations from 107 723 high‐quality cells derived from clinical tumor samples (Figure S1A and Table S1). Crucially, malignant cells and tumor associated macrophages (TAMs) emerged as the two most dominant cell populations, suggesting their core roles in tumor progression and recurrence. Malignant cells were further clustered into neural‐progenitor‐like (NPC), oligodendrocyte‐progenitor‐like (OPC), astrocyte‐like (AC), and mesenchymal‐like subpopulations (MES1 and MES2) based on canonical cell type markers (Figure 1B and Table S2). Within these specimens, MES1 subpopulations demonstrated the most complex functional profiles and the strongest epithelial‐mesenchymal transition (EMT) and fatty acid metabolism signature, being most pronounced in recurrent GBM samples (Figure S1B). We observed significant enrichment of the MES subpopulation, in recurrent disease (RD) GBM specimens (Figure 1C), where these cells predominantly accumulate within the hypoxic core regions of tumors (Figure 1D and Figure S1C). These results demonstrated that the perinecrotic hypoxic MES1 subpopulation serves as a critical driver of tumor recurrence in GBM.

**FIGURE 1:**
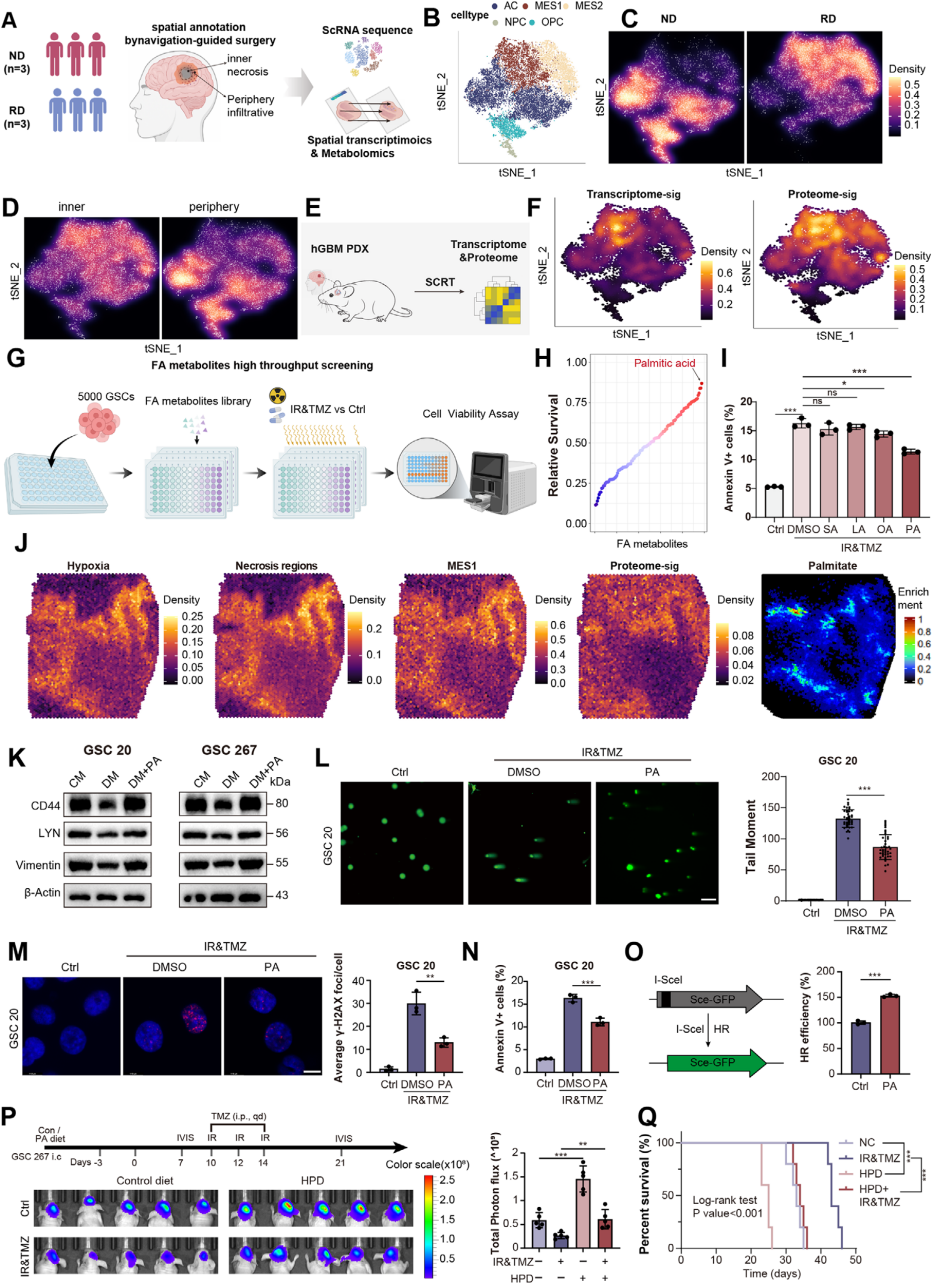
Multi‐omics analysis identifies palmitic acid as a key driver of GBM recurrence and therapeutic resistance. (A) Schematic illustration of sample collection and multi‐omics analysis. Tumor tissues were obtained from newly diagnosed (ND) and recurrent disease (RD) glioblastoma (GBM) patients. Inner necrotic and infiltrative peripheral regions were spatially annotated and subjected to single‐cell RNA sequencing (scRNA‐seq), spatial transcriptomics, and metabolomics profiling. (B) t‐SNE plot of scRNA‐seq data showing the distribution of major GBM‐associated cell populations. (C) Density plots of ND versus RD samples reveal distinct enrichment patterns of recurrent tumor–associated populations. (D) Density plots highlighting differences between tumor inner necrotic and infiltrative peripheral regions, showing spatially distinct cell distributions. (E) Schematic of the human GBM patient‐derived xenograft (PDX) model subjected to standard chemoradiotherapy (SCRT), followed by transcriptomic and proteomic profiling. (F) Density plots of Therapy resistance‐associated signatures (RS) derived from transcriptomic and proteomic datasets. (G) Flow chart for screening fatty acid metabolites that affect therapeutic resistance in GSCs. (H) Scatter plot depicting the relative viability of GSCs following chemoradiotherapy with pre‐administration of various fatty acids. (I) Quantification of Annexin V+ GSCs following chemoradiotherapy with various fatty acid treatments. Palmitic acid (PA) markedly attenuates therapy‐induced apoptosis in GSCs, n=3. (J) Spatial enrichment of hypoxia, necrosis, MES1 signatures, therapy resistance‐associated signatures, and palmitate enrichment in GBM. (K) Immunoblot analysis of MES markers in GSCs cultured in complete medium (CM) or dialyzed medium (DM) with or without palmitic acid supplementation. (L) Comet assays (left) and quantification of tail moment showing the effect of PA on chemoradiotherapy‐induced GSC DNA damage, n=40. Scale bar, 50 µm. (M) Immunofluorescence (IF) staining and quantification of γ‐H2AX foci in GSCs post‐chemoradiotherapy with or without PA treatment, n=3. Scale bar, 10 µm. (N) Flow cytometry quantification of apoptotic cells percent to assess the impact of PA on therapy resistance, n=3. (O) Schematic representation of the HR reporter (left). HR repair efficiency was measured in GSC 267 with or without PA treatment, n=3. (P) In vivo bioluminescence imaging of tumor‐bearing mice fed with control diet (Ctrl) or high‐palmitate diet (HPD) with or without chemoradiotherapy. Quantification of tumor photon flux is shown on the right, n=5. (Q) Kaplan‐Meier survival curves of tumor‐bearing mice under indicated treatment conditions, n=5. Data are presented as mean ± sd. *p < 0.05, **p < 0.01, ***p < 0.001 by Student's t‐test or one‐way ANOVA as appropriate.

To further validate this phenomenon, we established orthotopic patient‐derived xenograft models using human GBM patient‐derived xenograft (hGBM PDX) in immunodeficient mice. Mice treated with extended standard chemoradiotherapy (SCRT) developed therapy‐resistant tumors, showing reduced apoptosis and attenuated DNA damage post‐chemoradiotherapy (Figure S1D–G) versus control (Ctrl) group. This regimen selectively cleared therapy‐sensitive cells while enriching resistant populations. Subsequently, RNA transcriptome sequencing and proteomic profiling were performed on Ctrl and SCRT tumor cells to identify the key features associated with chemoradiotherapy resistance in GBM (Figure 1E). We observe that the upregulated transcriptome signature (transcriptome‐sig) and proteome signature (proteome‐sig) in chemoradioresistant tumor subpopulations predominantly map to the MES‐like subpopulations, with heightened prominence in the functionally more complex MES1 subpopulation (Figure 1F), indicating its central role in tumor recurrence. Further metabolic activity analysis revealed a marked elevation in fatty acid transport activity within SCRT group (Figure S2A and Table S3). In addition, scRNA functional enrichment analysis revealed significant enrichment of both MES1 program activity and fatty acid metabolic pathways (particularly the uptake and catabolism of long‐chain fatty acids) in recurrent GBM cells (Figure S2B). We further validated using an independent scRNA‐seq dataset that the MES subtype of GBM is characterized by enhanced fatty acid transport activity (Figure S2C,D). To further identify key regulatory fatty acids in GBM, we conducted metabolomic profiling and found that palmitic acid (PA), oleic acid (OA), stearic acid (SA), and linoleic acid (LA) were significantly enriched in GBM tissues (Figure S2E). We then established a fatty acid library and performed high‐throughput screening, which revealed that PA markedly enhanced the therapy resistance of glioma stem cells (GSCs) compared to other fatty acids (Figure 1G–I). Spatial transcriptomics and metabolomics confirm co‐enrichment of the MES1 tumor subpopulation, proteomics_sig and palmitate within hypoxia‐characterized pseudopalisading niches (Figure 1J), indicating that palmitate activities serve as a critical driver in GBM chemoradiotherapy resistance and recurrence.

### 2.2 SLC27A3‐Mediated PA Uptake Facilitates the MES Subtype and Therapy Resistance in GSCs

We next examined the relationship between palmitate availability and GBM MES transition. Validation studies using GSCs via BODIPY FL C16 pulse‐chase assays demonstrate time‐dependent potentiation of PA in MES GSCs compared to proneural (PN) GSCs (Figure S3A–C). Furthermore, spatial transcriptomic data demonstrated significant enrichment of long‐chain fatty acid uptake activity in regions densely populated with MES cells (Figure S3D). The solute carrier family 27 (SLC27) plays a crucial role in mediating palmitic acid transport (PAT), thereby facilitating its uptake and intracellular trafficking. To investigate the underlying mechanism driving elevated PAT in MES GSCs, we systematically knocked down individual SLC27 family using RNA interference. The results indicated that silencing SLC27A3 significantly reduced PAT activity in MES GSCs (Figure S3E). Consistently, SLC27A3 was upregulated in MES GBM tissues and MES GSC, correlating with poorer GBM patient survival, and its RNA and protein levels increase in SCRT tumor cells (Figure S3F–I). Collectively, these data suggested SLC27A3 as the principal molecular conduit for PA influx in MES GSCs. Furthermore, we found that PA promoted the maintenance of the MES subtype, as indicated by increased expression of MES markers including CD44, LYN, and Vimentin (Figure S4A), as well as promotion of invasive capacity, the latter being a fundamental trait of MES phenotypes, in a co‐culture model of tumor spheroids and normal rat brain organoids (Figure S4B). Critically, SLC27A3 knockdown reversed PA‐induced MES transition (Figure S4C). Additionally, MES GSCs cultured in PA‐deficient dialyzed medium (DM) exhibited loss of MES‐subtype markers and therapy resistance compared to their counterparts maintained in complete medium (CM). This phenotypic shift and associated therapy resistance were partially alleviated by exogenous PA supplementation (Figure 1K and Figure S4D). Therapy‐induced DNA double‐strand breaks (DSBs) triggered γH2AX foci accumulation, a hallmark of treatment resistance when rapidly resolved by enhanced DNA repair machinery in tumor cells [26]. We observed that PA treatment significantly mitigated chemoradiotherapy‐induced DNA damage in MES GSCs, as evidenced by reduced comet tail moments (Figure 1L and Figure S4E) and γH2AX foci formation (Figure 1M and Figure S4E), concomitant with reduced apoptosis (Figure 1N and Figure S4F). Given the critical role of homologous recombination (HR) in the repair of DNA double‐strand breaks, we employed a DR‐GFP reporter assay and found that PA significantly enhanced HR repair efficiency (Figure 1O). We further investigated its impact on glioma therapeutic resistance in vivo. Mice fed a palm oil‐rich diet exhibited accelerated tumor growth and reduced chemoradiotherapy efficacy compared to controls (Figure 1P,Q, and Figure S4G). Collectively, these data demonstrated that SLC27A3‐mediated PA uptake in GSCs potentiated MES and chemoradiotherapy resistance phenotypes, ultimately driving GBM recurrence.

### 2.3 Palmitoyl‐dependent Glycolysis Fuels MES Subtype and Therapy Resistance in GBM

PA is converted to palmitoyl‐CoA by long‐chain acyl‐CoA synthases, after which it can either enter mitochondria for β‐oxidation or serve as a substrate for protein palmitoylation [27, 28] (Figure 2A). To determine the dominant pathway, GSCs were treated with the β‐oxidation inhibitor Etomoxir or the palmitoyl‐transferase inhibitor 2‐bromopalmitate (2‐BP). Immunoblot analysis revealed that 2‐BP, but not Etomoxir, effectively abrogated PA‐induced upregulation of MES markers (Figure 2B), implicating palmitoylation as the primary mediator of the PA‐induced MES subtype. Moreover, 2‐BP substantially restored GSC apoptosis following therapy in PA‐treated cells, while Etomoxir exerted only marginal effects (Figure 2C and Figure S5A). Similarly, both the alkaline comet assay and γH2AX IF staining further confirmed that, while PA attenuated therapy‐induced DNA damage, this protective effect was significantly abrogated by 2‐BP treatment (Figure 2D,E, and Figure S5B,C). Collectively, these findings indicate that the pro‐tumorigenic and resistance‐enhancing effects of PA in GSCs are largely dependent on protein palmitoylation.

**FIGURE 2:**
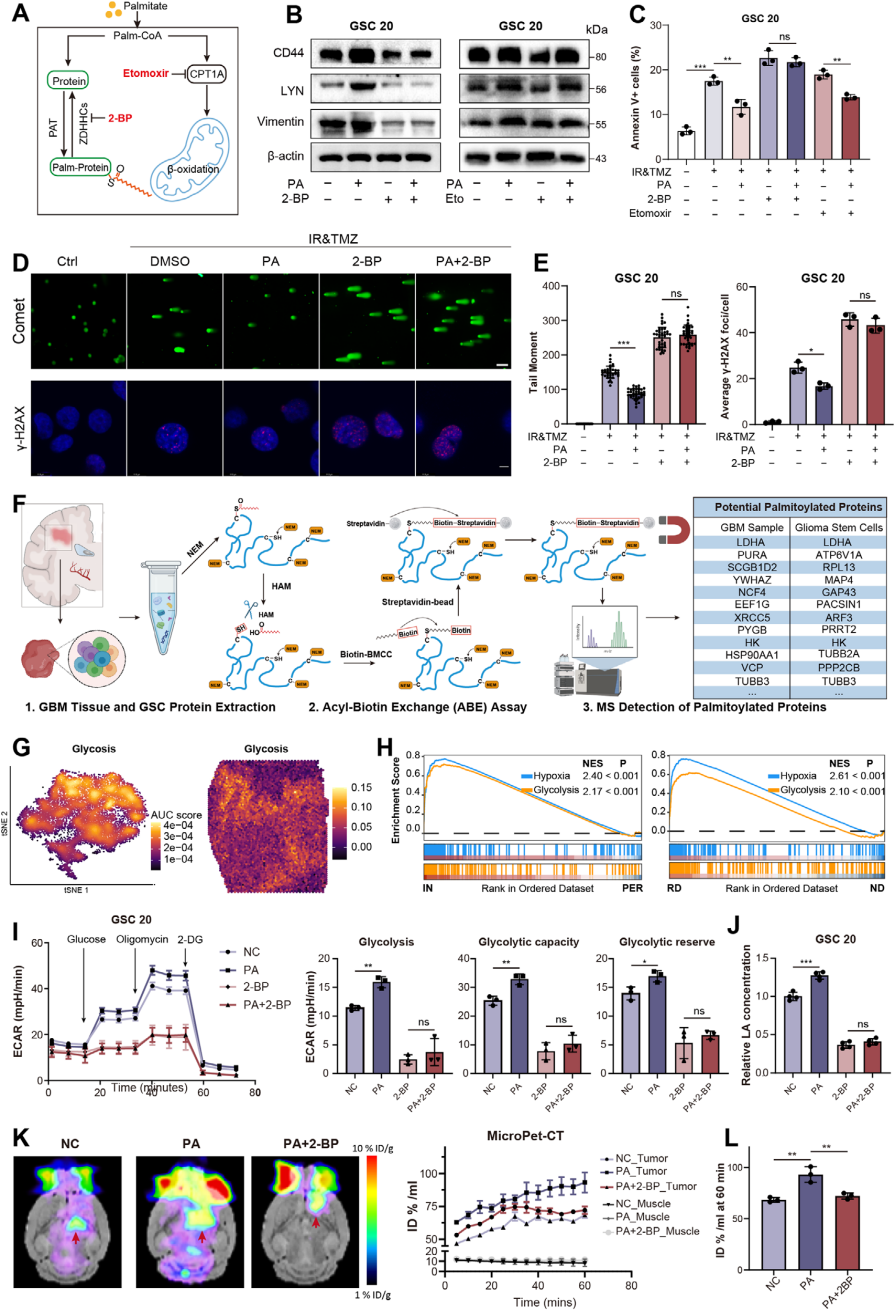
PA regulates GSC therapeutic resistance and glycolytic metabolism through protein palmitoylation. (A) Schematic illustration of the palmitoylation pathway in GSCs. PA is converted to palmitoyl‐CoA, which serves as a substrate for protein palmitoylation or transport to mitochondria. (B) Palmitoylation inhibitor (2‐BP) significantly inhibited PA‐induced upregulation of MES markers compared to FAO inhibitor (etomoxir). (C) Quantification of Annexin V+ apoptotic cells in GSCs under indicated treatments following chemoradiotherapy, n=3. (D) Representative images of alkaline comet assay (top, Scale bar, 50 µm) and γ‐H2AX IF staining (bottom, Scale bar, 5 µm) in GSC 20 after indicated treatments. (E) The quantification of average γ‐H2AX foci per cell (left, n=3) and comet tail moment (right, n=40) under indicated conditions. (F) Schematic workflow of the Acyl Biotin Exchange (ABE) assay for the detection of palmitoylated proteins in GBM tissues and GSCs, followed by LC‐MS/MS identification. (G) Density plots showing glycolysis activity across single‐cell transcriptomes and spatial transcriptomes. (H) Gene set enrichment analysis (GSEA) demonstrating significant enrichment of glycolysis and hypoxia signatures in GBM samples, with higher enrichment observed in necrotic inner (IN) regions versus infiltrative periphery (PER) (left) and in recurrent disease (RD) versus newly diagnosed (ND) tumors (right). (I) Seahorse extracellular acidification rate (ECAR) analysis of glycolytic function in GSC 20 treated with PA and/or 2‐BP. Left, ECAR time course; right, quantification of glycolysis, glycolytic capacity, and glycolytic reserve, n=3. (J) Quantification of lactate LA production in GSC 20 under indicated treatments, n=3. (K) Representative micro‐PET CT images (left) and time‐activity curves (right) showing ^18^F‐FDG uptake in intracranial tumor‐bearing mice. (L) Quantification of maximum standardized uptake value (SUVmax) in tumors from micro‐PET CT analysis, n=3. Data are presented as mean ± sd. *p < 0.05, **p < 0.01, ***p < 0.001 by Student's *t‐*test or one‐way ANOVA as appropriate.

To investigate the downstream targets and functional consequences of palmitoylation, we carried out acyl‐biotin exchange (ABE) experiments combined with mass spectrometry using both MES GSCs and GBM tumor tissues (Figure 2F and Table S4). This analysis identified palmitoylated proteins consistently modified in both GSCs and GBM tumor tissues. Enrichment analysis revealed that palmitoylated proteins were significantly associated with the glycolytic pathway (Figure S5D). Hypoxia, a hallmark of pseudopalisading necrotic regions, compels tumor cells to rely heavily on glycolytic energy production to adapt to this hostile microenvironment [29, 30]. Additionally, both our scRNA and ST analysis consistently demonstrated enhanced glycolytic activity in hypoxic pseudopalisading necrotic regions and areas enriched with the MES1 cell subpopulation (Figure 2G). GSEA further revealed significantly enhanced hypoxia‐associated glycolytic activity in both necrotic inner regions and recurrent tumor populations (Figure 2H). And seahorse analysis of extracellular acidification rate (ECAR) revealed that MES GSCs exhibited markedly higher glycolytic capacity and reserve compared to PN GSCs (Figure S5E). We next assessed whether PA regulates glycolytic processes. Glycolytic stress tests revealed that PA treatment significantly elevated the ECAR, indicating enhanced glycolytic flux in GSC 20 and GSC 267, whereas 2‐BP effectively reversed this effect (Figure 2I and Figure S5F). Correspondingly, PA treatment also increased intracellular lactate levels, which were attenuated by 2‐BP co‐treatment (Figure 2J and Figure S5G). Additionally, SCRT tumor cells exhibited higher glycolytic activity compared to Ctrl tumor cells, whereas treatment with 2‐BP suppressed glycolysis in both groups (Figure S5H). To further evaluate the impact of PA‐induced palmitoylation on glycolysis in vivo, we employed ^18^F‐FDG PET/CT imaging to assess glycolysis in xenograft models. The results demonstrated that PA‐enriched diet markedly increased glucose uptake in tumors, whereas co‐administration of 2‐BP significantly attenuated this effect (Figure 2K,L). Collectively, these findings demonstrate that MES GSCs possess greater metabolic flexibility and an enhanced ability to upregulate glycolytic flux under metabolic stress, which may contribute to their increased survival, adaptability, and resistance to therapeutic interventions.

### 2.4 ZDHHC12 Contributes to MES Subtype and Chemoradiotherapy Resistance in GBM

Protein palmitoylation is catalyzed by zinc finger DHHC domain‐containing palmitoyl‐transferases (ZDHHCs), a family comprising 23 members in mammals [31, 32]. In terms of the important role of palmitoylation on glycolysis and MES subtype, we initially performed correlation and survival prognosis analysis based on the TCGA GBM cohort. We noted that ZDHHC12 was highly positively correlated with glycolysis, MES subtype, and poor prognosis in the GBM dataset (Figure S6A–D). Further analysis of sequencing results from the SCRT and Ctrl tumor cells revealed that the mRNA expression levels of ZDHHC1, 3, 5, 12, 14, 16, and 24, as well as the protein levels of ZDHHC12 and ZDHHC13, were significantly upregulated in the SCRT tumor cells (Figure S6E–G). Knockdown of ZDHHC12 in SCRT tumor cells enhanced their sensitivity to therapy, whereas overexpression of ZDHHC12 in Ctrl tumor cells promoted therapy resistance. In contrast, manipulation of ZDHHC13 expression did not result in a significant change in therapy sensitivity (Figure S6H,I). Furthermore, analysis of public GBM datasets indicated that high ZDHHC12 expression is associated with reduced overall survival (Figure S6J,K). ScRNA‐seq analysis further revealed elevated ZDHHC12 expression specifically in the MES1‐GBM subpopulation (Figure 3A). Pseudotime trajectory analysis using Monocle2 indicated progressive upregulation of ZDHHC12 expression during tumor progression, with notably stronger expression in recurrent GBM cells (Figure 3B). And the IHC staining suggested that ZDHHC12 protein expression increased with elevated glioma grade (Figure 3C). Moreover, knockdown of ZDHHC12 significantly attenuated therapy resistance in MES GSCs (Figure 3D and Figure S7A) and suppressed the expression of MES signature markers (Figure 3E). Simultaneously, rescue experiments demonstrated that ZDHHC12‐knockdown cells exhibited substantially diminished responses to PA administration compared to control cells (Figure 3F,G, and Figure S7B). Collectively, these data indicated that the palmitoyl‐transferase ZDHHC12 mediates MES transition and therapy resistance in GSCs through palmitoylation‐dependent pathways.

**FIGURE 3:**
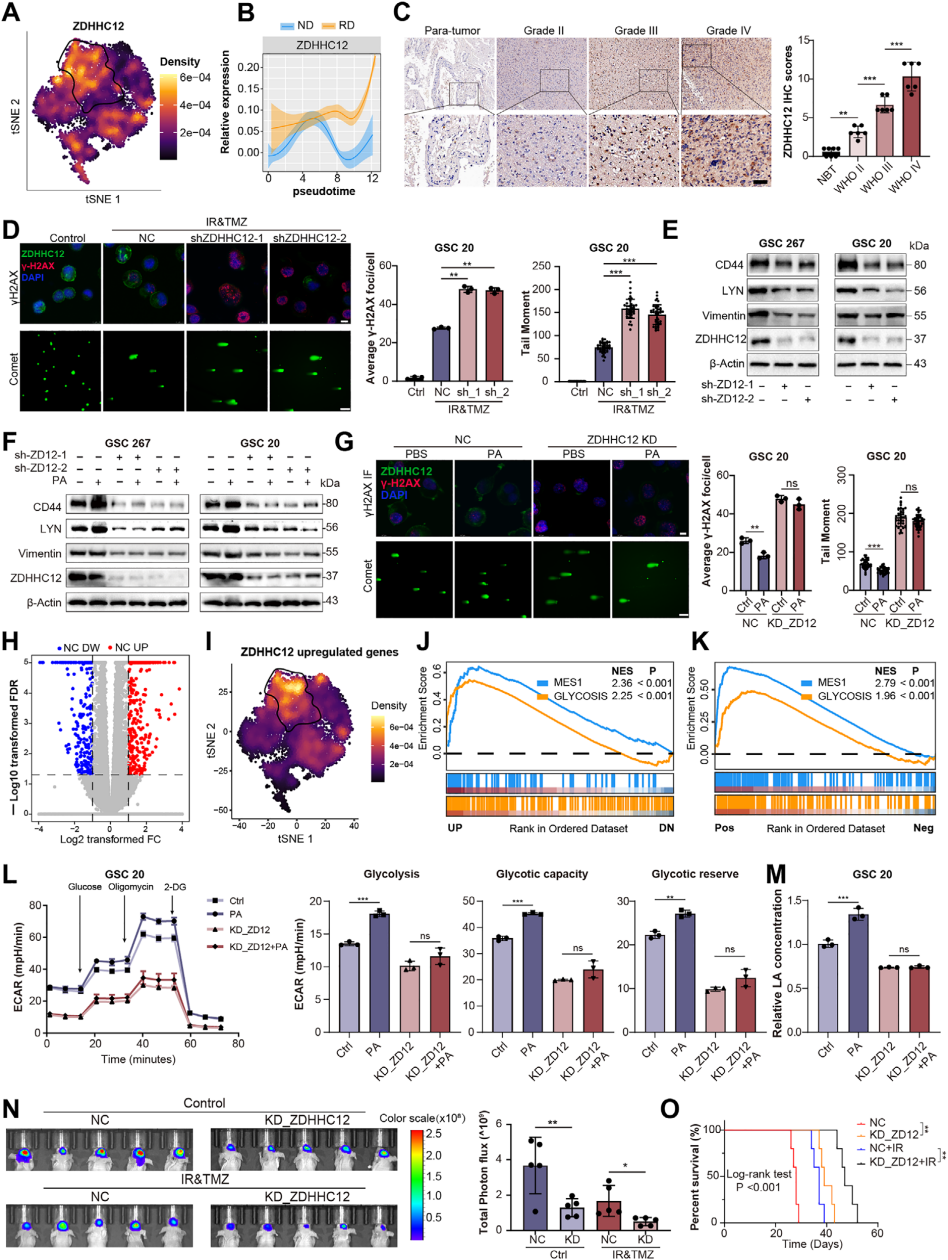
Identification of ZDHHC12 as a critical regulator of glycolytic reprogramming and therapy resistance in MES GSCs. (A) t‐SNE density map showing the distribution of ZDHHC12 expression across GBM single‐cell transcriptomes. (B) Pseudotime trajectory analysis of ZDHHC12 expression in newly diagnosed (ND) and recurrent disease (RD) GBM, demonstrating elevated expression in RD tumors. (C) Representative immunohistochemistry (IHC) images and scoring of ZDHHC12 in glioma tissues of different grades and adjacent non‐tumor tissue (Para‐tumor). Scale bar, 50 µm. (D) IF staining (top, Scale bar, 10 µm) and alkaline comet assay (bottom, Scale bar, 50 µm) for γ‐H2AX in GSC 20 transduced with control or ZDHHC12 shRNA, with or without chemoradiotherapy. Quantifications of γ‐H2AX foci (n=3) and tail moment (n=40) are shown on the right panel. (E) Knockdown of ZDHHC12 inhibited the protein expression of MES markers in GSC 20 and GSC 267. (F) Immunoblot analysis showing the effects of PA intervention on the expression of MES marker in control or ZDHHC12 knockdown GSCs. (G) γ‐H2AX IF (top, Scale bar, 10 µm) and comet assay (bottom, Scale bar, 50 µm) of ZDHHC12 knockdown or control GSCs after therapy with or without PA. Quantification of γ‐H2AX foci (n=3) and tail moment (n=40) are shown on the right panel. (H) Volcano plot depicting significantly altered gene expression profiles following ZDHHC12 knockdown in GSC 267. (I) t‐SNE density map demonstrating that the expression pattern of ZDHHC12‐upregulated genes significantly overlaps with the MES1 subset region. (J) GSEA showing that ZDHHC12‐upregulated genes are significantly enriched in MES1 and glycolysis signatures. (K) GSEA based on scRNA‐seq data demonstrating that ZDHHC12 positively correlated genes are significantly enriched in MES1 and glycolysis signatures. (L) Seahorse extracellular acidification rate (ECAR) analysis in GSC 20 and GSC 267 under indicated conditions. ZDHHC12 knockdown suppresses PA‐induced glycolysis, glycolytic capacity, and glycolytic reserve, n=3. (M) The relative concentration of lactate under the indicated treatment conditions, n=3. (N) In vivo bioluminescence imaging and quantification of total photon flux in mice orthotopically implanted with control or ZDHHC12 knockdown GSC 267 cells, with or without chemoradiotherapy, n=5. (O) Kaplan‐Meier survival analysis demonstrates that knockdown of ZDHHC12 in GSCs significantly enhances the survival benefit conferred by therapy, n=5. Data are presented as mean ± sd. *p < 0.05, **p < 0.01, ***p < 0.001 by Student's t‐test or one‐way ANOVA as appropriate.

To elucidate the downstream effects of ZDHHC12, transcriptomic analysis revealed that genes significantly downregulated upon ZDHHC12 knockdown were prominently enriched in the MES1 subpopulation. (Figure 3H,I). GSEA further demonstrated that knockdown of ZDHHC12 markedly suppressed MES transition and glycolytic signatures (Figure 3J). Consistently, correlation analysis of scRNA data identified genes positively correlated with ZDHHC12 to be functionally implicated in both MES transition and glycolytic activity (Figure 3K). Seahorse glycolytic stress test further demonstrated that PA‐induced enhancement of glycolysis, glycolytic capacity, and glycolytic reserve was abolished by ZDHHC12 knockdown in MES GSCs (Figure 3L and Figure S7C). Consistent results were obtained from measurements of lactic acid content (Figure 3M and Figure S7D). Metabolic rewiring toward glycolysis, accompanied by a reduction in OXPHOS‐derived mitochondrial ROS, may promote the survival of GSCs following chemoradiotherapy [33]. Therefore, we assess the regulation of OXPHOS by ZDHHC12 by mitochondrial stress assays. Knockdown of ZDHHC12 significantly increased the OCR of GSC 20 and GSC 267, accompanied by a significant increase in ROS induced by chemoradiotherapy (Figure S8A–C). These findings suggested that inhibition of ZDHHC12‐mediated palmitoylation leads to elevated mitochondrial respiration and heightened oxidative stress following chemoradiotherapy (Figure S8D).

To evaluate the effect of ZDHHC12 on the therapy sensitivity of GSCs in vivo, we established xenograft models using control and ZDHHC12‐knockdown GSC 267 followed by chemoradiotherapy. The xenograft models established with ZDHHC12‐KD GSCs showed significantly reduced tumor growth under both control and therapy conditions (Figure 3N and Figure S9A). Survival analysis revealed prolonged survival in mice bearing ZDHHC12‐knockdown tumors, particularly following therapy (Figure 3O). Hematoxylin and eosin (H&E) staining, along with IHC staining of ZDHHC12 and γH2AX, further confirmed diminished tumor burden and enhanced DNA damage in the ZDHHC12 knockdown group under chemoradiotherapy (Figure S9B). Collectively, these findings demonstrate that ZDHHC12 drives glycolytic metabolic reprogramming and contributes to the therapy resistance of GSCs in vitro and in vivo.

### 2.5 ZDHHC12‐Mediated LDHA Palmitoylation at Cys163 Drives MES Subtype and Therapy Resistance in GBM

To identify potential palmitoylated substrates regulated by ZDHHC12, we performed co‐immunoprecipitation (Co‐IP) assays to screen for ZDHHC12‐interacting proteins. By intersecting these candidates (Table S5) with palmitoylated proteins previously identified in GSCs and GBM tissues (Figure 2F), we identified six overlapping targets (Figure 4A). Among them, VDAC1, PKM2, SLC25A5 and LDHA are closely related to metabolic reprogramming and mitochondrial/glycolytic regulation, which are key biological features associated with the MES subtype of GBM [34, 35, 36, 37]. We therefore prioritized these four candidates for ABE‐based validation of ZDHHC12‐dependent palmitoylation. The results showed that ZDHHC12 knockdown reduced the palmitoylation levels of all four candidates to varying degrees, with the most pronounced decrease observed for LDHA, followed by a moderate reduction in PKM2, whereas VDAC1 and SLC25A5 were only minimally affected (Figure S10A). To further evaluate the functional contribution of LDHA and PKM2, we performed rescue experiments. Knockdown of LDHA significantly suppressed PA‐induced upregulation of MES markers and therapy‐resistance of GSC, whereas PKM2 knockdown had a negligible effect (Figure 4B,C), indicating LDHA as a critical effector of palmitoylated targeted protein. Co‐IP assays further confirmed the interaction between ZDHHC12 and LDHA (Figure S10B). We next detected the palmitoylation of LDHA using ABE assay and observed that LDHA could be palmitoylated, whereas 2‐BP treatment or knockdown of ZDHHC12 reduced the levels of palmitoylation on LDHA (Figure 4D,E and Figure S10C). We further assessed the palmitoylation levels of endogenous LDHA in clinical specimens, including normal brain tissue and glioma samples of varying grades. LDHA palmitoylation levels progressively increased with glioma grade, correlating with elevated ZDHHC12 expression (Figure 4F). Collectively, these data established LDHA as a functionally significant palmitoylation substrate of ZDHHC12.

**FIGURE 4:**
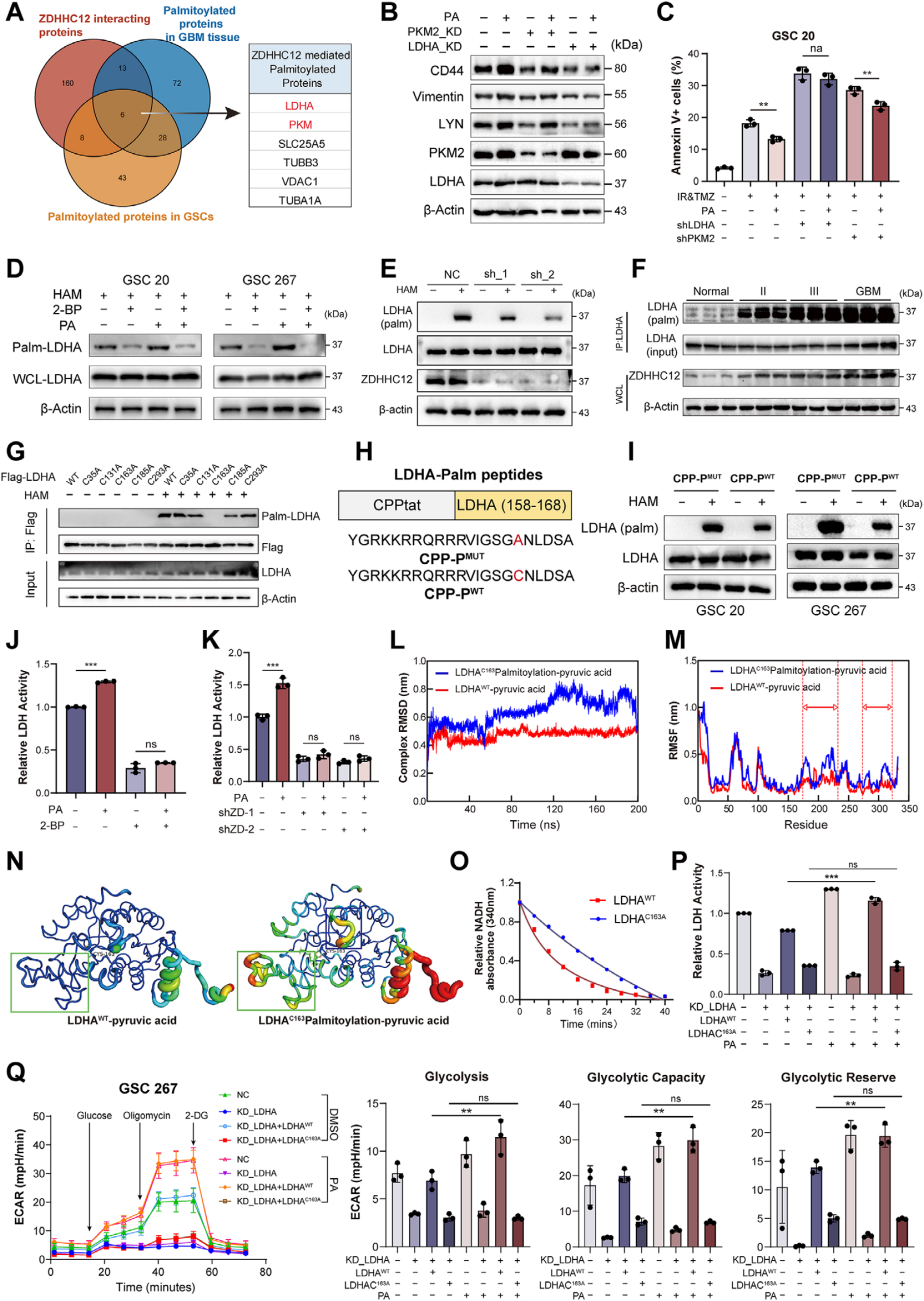
PA enhances LDHA enzymatic activity through ZDHHC12‐mediated palmitoylation at Cys163. (A) Venn diagram identifying ZDHHC12 co‐immunoprecipitated (co‐IP) proteins, palmitoylated proteins in GBM tissue and GSCs. The overlap highlights key ZDHHC12‐mediated palmitoylated targets associated glycolysis, including LDHA and PKM2. (B) Immunoblot analysis showing that knockdown of LDHA abrogates PA‐induced upregulation of MES markers in GSCs, whereas knockdown of PKM2 has minimal effect. (C) Quantification of Annexin V+ apoptotic cells showing that LDHA knockdown, but not PKM2 knockdown, reverses the anti‐apoptotic effect of PA, n=3. (D) ABE assay showing that PA increases LDHA palmitoylation, which is blocked by 2‐BP. (E) Knockdown of ZDHHC12 suppressed LDHA palmitoylation in GSC 267 assessed by ABE assay. (F) Immunoblot analysis of LDHA palmitoylation and ZDHHC12 expression in clinical samples. (G) Immunoblot analysis of LDHA palmitoylation by ABE assay in HEK293T cells transfected with plasmids encoding either wild‐type LDHA‐Flag or the indicated mutants. (H,I) Schematic and validation of LDHA palmitoylated peptide (CPP‐palm) for functional inhibition of LDHA palmitoylation in GSC 20 and GSC 267. (J,K) LDH activity assessment of GSC 20 in the indicated groups. (L,M) Molecular dynamics simulations assessing the structural stability of wild‐type LDHA versus C163‐palmitoylated LDHA, evaluated through RMSD and RMSF metrics. (N) B‐factor analyses illustrating conformational differences caused by palmitoylation at Cys163. (O) In vitro LDHA activity assay with Flag‐tagged WT and C163A mutants; NADH absorbance measured at 340 nm, n=3. (P) LDH activity assay of GSC 20 cells to evaluate the effect of LDHA^C163A^ mutation on enzymatic activity, n=3. (Q) Seahorse extracellular flux analysis evaluating glycolytic function in GSCs with LDHA knockdown, followed by rescue with either wild‐type LDHA or the C163 mutant, n=3. Data are presented as mean ± sd. *p < 0.05, **p < 0.01, ***p < 0.001 by Student's *t*‐test or one‐way ANOVA as appropriate.

As a post‐translational modification, palmitoylation covalently anchors palmitoyl groups to cysteine residues via thioester bonds, which directly modulate substrate stability, subcellular trafficking, and enzymatic activity [38, 39, 40]. Through screening, we identified that LDHA contains five highly conserved cysteine residues (Figure S10E). To identify the critical palmitoylation site on LDHA, we generated a series of LDHA point mutants in which specific cysteine residues were substituted with alanine (C‐A) (Figure S10F). We found that mutating all potential sites completely abolished LDHA palmitoylation, whereas reintroducing cysteine at position 163 restored it, thereby identifying Cys163 as the major palmitoylation site of LDHA (Figure S10G). Additionally, single‐point mutant validation revealed that the palmitoylation level of the LDHA^C163A^ mutant was significantly lower than that of wild‐type (WT) LDHA, further confirming the critical role of Cys163 in LDHA palmitoylation (Figure 4G). To further confirm the palmitoylation site of LDHA, we constructed a CPP‐fused competing peptide encompassing the LDHA amino acid sequence 158–168 containing the palmitoylation motif (CPP‐P^WT^), along with its site‐specific mutant variant (CPP‐P^MUT^). Notably, treatment with CPP‐P^WT^ markedly reduced LDHA palmitoylation levels compared to CPP‐P^MUT^, supporting the critical role of Cys163 in mediating LDHA palmitoylation (Figure 4H,I). Rescue assays in LDHA‐knockdown GSCs showed that while LDHA^WT^ restored MES marker expression, the C163A mutant failed to do so (Figure S10H), highlighting the functional role Cys163 in mediating LDHA palmitoylation. To determine whether C163 mutation affects ZDHHC12‐LDHA binding, we further performed ZDHHC12 Co‐IP and PLA assays. LDHA^C163A^ showed comparable association and proximity with ZDHHC12 relative to LDHA^WT^ (Figure S10I,J), indicating that Cys163 mutation impairs LDHA palmitoylation without disrupting ZDHHC12‐LDHA interaction.

### 2.6 LDHA Cys163 Palmitoylation Modulates Its Structure, Enzymatic Activity and Glycolytic Reprogramming

It has been shown that palmitoylation play a key regulatory role by influencing protein expression, enzyme activity, and extracellular secretion [31, 38, 40, 41]. We first explored the regulatory mechanisms of endogenous palmitoylated LDHA. The LDHA protein expression and subcellular localization were not significantly altered by PA treatment or 2‐BP (Figure S11A,B). We found that PA promoted LDHA enzymatic activity. Notably, rescue experiments demonstrated that inhibition of palmitoylation by 2‐BP or knockdown of ZDHHC12 significantly reversed this stimulatory effect (Figure 4J,K). We further performed an in vitro LDHA enzymatic assay to evaluate the direct effect of Cys163 palmitoylation on LDHA activity. LDHA^WT^ showed a faster decline in NADH absorbance at 340 nm compared with LDHA^C163A^ (Figure 4O). In addition, Palmitoyl‐CoA increased LDHA^WT^ activity, elevated Vmax and reduced Km, but failed to affect LDHA^C163A^ (Figure S11C), indicating that Cys163 palmitoylation enhances LDHA catalytic efficiency and pyruvate binding.

To probe the structural and functional consequences of LDHA palmitoylation at site Cys163, we performed molecular dynamics (MD) simulations comparing wild‐type and palmitoylated LDHA‐pyruvate complexes (Figure S11D). A 200 ns simulation revealed distinct differences in structural stability and dynamic behavior between the two complexes. The root mean square deviation (RMSD) analysis showed that the WT complex remained relatively stable throughout the simulation, with RMSD values fluctuating between 0.4 and 0.6 nm. In contrast, the LDHA^C163^‐Palmitoylation complex exhibited a progressive increase in RMSD, reaching up to 0.8 nm after 120 ns, indicating reduced structural stability and increased conformational fluctuations (Figure 4L). Consistent with this, root mean square fluctuation (RMSF) and B‐factor analyses revealed markedly enhanced flexibility in the region surrounding Cys163, as well as increased dynamic activity in the substrate‐binding domain (Figure 4M,N). These results suggest that palmitoylation introduces local structural perturbations that propagate to broader regions of the LDHA, potentially impacting its catalytic behavior.

Despite decreased structural stability, MM/PBSA analysis revealed enhanced binding affinity of LDHA for pyruvate upon palmitoylation (the total binding free energy (ΔG_total) decreased from −14.04 kcal/mol in the WT complex to −15.16 kcal/mol in the palmitoylated complex, driven primarily by strengthened van der Waals interactions facilitated by the hydrophobic palmitoyl moiety (Figure S11E–H). These data indicate that palmitoylation may stabilize the enzyme–substrate interaction through favorable nonpolar contacts, despite introducing greater conformational flexibility.

To validate the functional role of LDHA C163 palmitoylation, we generated LDHA‐knockdown GSCs and reconstituted them with either wild‐type or C163A‐mutant LDHA plasmids, enabling the evaluation of palmitoylation‐dependent effects on tumor cell behavior. LDHA silencing reduced LDH enzyme activity, and the recovery of LDHA^WT^ expression exhibited restored effects, while LDHA^C163A^ failed to rescue LDHA enzyme activity in response to PA (Figure 4P). Seahorse analysis was performed to assess the role of LDHA Cys163 palmitoylation in glycolysis. In LDHA‐knockdown GSC 267, re‐expression of LDHA^WT^ restored glycolytic activity, glycolytic capacity, and glycolytic reserve upon PA treatment, whereas the LDHA^C163A^ failed to do so (Figure 4Q). These results indicate that Cys163 palmitoylation is essential for PA‐induced glycolytic reprogramming in GSCs.

### 2.7 ZDHHC12‐Mediated LDHA Palmitoylation Promotes Its Exosomal Sorting in Hypoxic MES‐GSCs

MES‐GBM cells exhibit intrinsic hypoxic properties (Figure 1D and Figure S1B), further corroborated by spatial transcriptomics (ST) data revealing their extensive localization within tumor hypoxic niches (Figure 1J). Our proteomic analysis detected pronounced enrichment of LDHA in exosomes derived from hypoxia‐exposed GSCs (Figure 5A), validated by immunoblot analysis (Figure 5C). Building on established evidence that palmitoylation directly governs exosomal protein sorting [42], we propose that PA facilitates exosomal packaging of LDHA through this post‐translational modification. Crucially, Duolink proximity ligation immunoblot assays demonstrated that hypoxia enhanced ZDHHC12‐LDHA binding capacity (Figure 5B and Figure S10D). And PA promoted LDHA enrichment in exosomes without significantly altering exosomal morphology or size (Figure S12A,B), while ZDHHC12 knockdown attenuated PA‐induced LDHA accumulation in exosomal fractions (Figure 5D). And mutation of LDHA palmitoylation sites (Cys163) profoundly impaired its exosomal sorting (Figure S12C). In summary, PA promoted exosomal LDHA sorting via ZDHHC12‐mediated palmitoylation.

**FIGURE 5:**
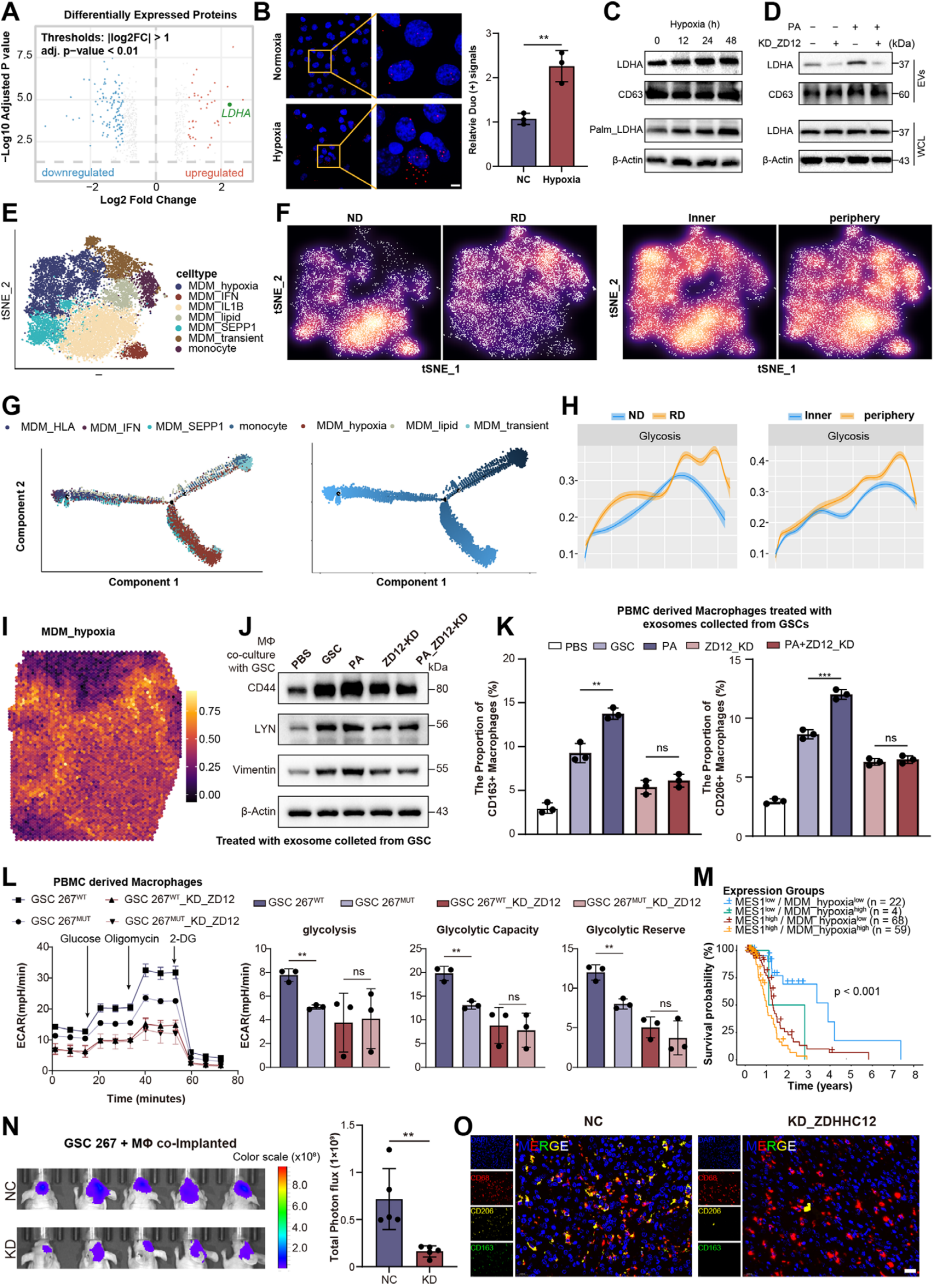
PA promotes the polarization of immunosuppressive macrophages via exosome‐mediated LDHA transfer. (A) Volcano plot of proteomic profiling highlighting differentially expressed proteins, with LDHA significantly upregulated. (B) Detection of ZDHHC12 and LDHA binding (red dots) by Duolink assay. Scale bar, 10 µm. (C) Immunoblot analysis showing increased LDHA and palmitoylated LDHA in extracellular vesicles (EVs) under hypoxic conditions. (D) Immunoblot of EVs and whole‐cell lysates (WCL) showing that PA enhances LDHA palmitoylation and secretion, which is reduced upon ZDHHC12 knockdown. (E) t‐SNE plot of single‐cell RNA‐seq data showing the distinct macrophage subclusters. (F) Density plots comparing macrophage distributions in ND versus RD tumors and between inner necrotic and peripheral infiltrative regions. (G) Pseudotime trajectory analysis revealing the differentiation of monocytes into macrophage subtypes. (H) Glycolysis scores of macrophages, showing higher activity in RD versus ND tumors (left) and in inner versus peripheral tumor regions (right). (I) The density maps of MDM_hypoxia signature enrichment overlap with regions previously identified as enriched for glycolysis and therapeutic resistance signatures. (J) Macrophages were first primed with GSC‐derived exosomes from the indicated treatment groups for 48 h, followed by co‐culture with naïve GSCs in a transwell system for 72 h. GSCs were then collected for immunoblot analysis of MES marker expression. (K) Quantification of the proportion of CD163^+^ and CD206^+^ macrophages in PBMC‐derived macrophages treated with different GSC‐derived exosomes, n=3. (L) Seahorse analysis of ECAR in PBMC‐derived macrophages exposed to exosomes from GSC 267 as indicated treatment, n=3. (M) Kaplan‐Meier survival analysis of GBM patients stratified by MES1 and MDM_hypoxia expression levels. (N) Bioluminescence imaging with quantification of total photon flux in mice co‐implanted with GSC 267 cells and macrophages, n=5. (O) IF staining of tumor sections showing decreased infiltration of immunosuppressive macrophages in ZDHHC12 knockdown tumors compared with NC. Data are presented as mean ± sd. *p < 0.05, **p < 0.01, ***p < 0.001 by Student's t‐test or one‐way ANOVA as appropriate.

Emerging evidence identifies tumor cell‐TAM interactions as critical mediators of GBM therapy resistance, with MES‐GBM orchestrating both TAM recruitment and immunosuppressive polarization [9, 43, 44]. The specific TAM subcluster engaged by MES‐GBM and molecular determinants of this interplay require systematic elucidation. Based on canonical cell‐type markers, TAMs were further clustered into myeloid‐derived macrophages (MDMs) and tissue‐resident microglias (MGs) (Figure S12D). Intriguingly, our data revealed significant enrichment of MDMs within hypoxic core regions and recurrent GBM specimens (Figure S12E,F). Spatial mapping detected pronounced MDM enrichment in hypoxic niches, coupled with hypoxia‐glycolysis pathway activation (Figure S12G). Functional enrichment analyses demonstrated that these MDMs exhibit characteristic hypoxia‐associated glycolytic signatures (Figure S12H,I). We proposed the MES1‐MDM crosstalk axis as a key orchestrator of recurrence dynamics. Further subclustering of MDMs resolved seven transcriptionally distinct subtypes (Figure 5E and Table S6), thereby specifying the intratumoral heterogeneity within these tumor‐infiltrating myeloid populations. Specifically, the hypoxia_MDM subcluster manifested a pronounced hypoxia‐responsive transcriptional program. This signature was characterized by upregulation of hypoxia activity, concomitant enhancement of glycolytic and pro‐angiogenic activities, and acquisition of epithelial‐to‐mesenchymal transition (EMT) traits (Figure S13A,B). Notably, these molecular signatures demonstrated elevated enrichment in recurrent tumors and tumor core regions (Figure S13A,B). Critically, MDM‐hypoxia subpopulation was significantly enriched in recurrent tumors and tumor core regions (Figure 5F and Figure S14A). Building upon pseudotime trajectory analysis, MDM‐hypoxia was identified at the terminal differentiation state of monocytic lineages (Figure 5G), with progressively enhanced glycolytic activity correlating with advancing tumor malignancy, particularly prominent in recurrent tumors and hypoxic core regions (Figure 5H). Critically, ST profiling reveals robust co‐localization of MDM‐hypoxia with MES1‐GBM subtypes and palmitate accumulation (Figure 5I). This spatial concordance implicates palmitate as a pivotal driver of intercellular crosstalk within the MES1‐MDM‐hypoxia niches.

### 2.8 Exosomal Palmitoylated LDHA Drives Macrophage Glycolytic Reprogramming and Reciprocal Macrophage–GBM Crosstalk

Next, we investigated whether PA induces MDM‐hypoxia subset formation by promoting packaging of the glycolytic rate‐limiting enzyme LDHA into exosomes for targeted delivery to MDMs. Single‐cell analysis revealed elevated transcriptional levels of immunosuppressive markers CD163/CD206 in recurrent tumor specimens, with heightened expression specifically observed in the low oxygen‐MDM subclass (Figure S14B). To dissect GSC‐macrophage crosstalk, we established a co‐culture system (Figure S14C). Functional assays using PBMC‐derived macrophages revealed that GSC‐derived exosomes drove macrophage polarization toward immunosuppressive CD163+/CD206+ phenotypes, and this effect was also potentiated by PA preconditioning but abrogated upon ZDHHC12 knockdown in GSCs (Figure 5K and Figure S14D). Furthermore, exosomes derived from LDHA^WT^‐reconstituted GSCs significantly promoted immunosuppressive macrophage polarization, whereas exosomes from LDHA^C163A^‐expressing cells abrogated this effect (Figure S14E). Notably, Seahorse metabolic analysis demonstrated that LDHA^C163A^ exosomes exhibited markedly impaired capacity to enhance macrophage glycolysis compared to LDHA^WT^ counterparts. Crucially, ZDHHC12 knockdown significantly attenuated the enhancement of macrophage glycolysis mediated by exosomes derived from LDHA^WT^ reconstituted GSCs. (Figure 5L), establishing Cys163 palmitoylation as essential for exosome‐mediated macrophage reprogramming.

In turn, exosome‐primed macrophages reciprocally promoted MES‐GSC remodeling (Figure 5J and Figure S14C), suggesting a bidirectional crosstalk between GSCs and macrophages. To further dissect the macrophage‐derived signals underlying this feedback regulation, we examined inflammatory cytokine expression in exosome‐treated macrophages. GSC‐derived exosomes markedly increased the expression of IL‐1β, IL‐6, TNF‐α and TGF‐β, among which IL‐1β showed the most prominent induction; this effect was further enhanced by PA‐preconditioned GSC exosomes but attenuated by ZDHHC12 knockdown (Figure S14F). Consistently, ELISA confirmed increased secretion of IL‐1β from PBMC‐derived macrophages after treatment with PA‐GSC exosomes, whereas ZDHHC12 knockdown reduced IL‐1β release (Figure S14G).

We next investigated whether macrophage‐derived IL‐1β could feed back to regulate PA uptake in GSCs. IL‐1β treatment increased p65 phosphorylation and SLC27A3 expression in GSC20 and GSC267 cells in a dose‐dependent manner at 0, 10 and 20 ng/mL (Figure S14H). Pharmacological inhibition of NF‐κB signaling with BMS‐345541 markedly suppressed IL‐1β‐induced p65 phosphorylation and SLC27A3 upregulation (Figure S14I). Functionally, IL‐1β enhanced palmitic acid uptake in GSCs, whereas BMS‐345541 largely abolished this effect (Figure S14J,K). These data suggest that exosome‐reprogrammed macrophages promote GSC palmitic acid uptake through an IL‐1β–NF‐κB–SLC27A3 feedback axis.

Clinically, patients with high proportions of both MES1‐GBM cell and hypoxia‐MDM had shorter OS than did those with lower proportions of both cell subpopulations (Figure 5M). Moreover, intracranial co‐implantation of ZDHHC12‐depleted MES‐GSCs with human PBMC‐derived macrophages revealed that palmitoylation deficiency attenuates tumor growth (Figure 5N) and reduces CD163+/CD206+ immunosuppressive macrophage infiltration (Figure 5O), concurrently improving survival outcomes (Figure S14M). Notably, we obtained similar results in immunocompetent mice (Figure S14L,M). Collectively, these results indicated that PA potentiated immunosuppressive MDM‐hypoxia subset formation via ZDHHC12‐mediated palmitoylation of LDHA at Cys163, enabling its exosomal packaging for targeted metabolic reprogramming of macrophages toward a protumorigenic glycolytic state.

### 2.9 Rutin Blocks Palmitoylation Site of LDHA, Enhancing Therapeutic Sensitivity of GBM

Given the pivotal role of palmitoylated LDHA in sustaining therapeutic resistance of MES GSCs and promoting an immunosuppressive TME, we performed a structure‐based virtual screening centered on the palmitoylation site at LDHA^C163^ to molecularly dock a library of approximately 120 000 small molecules (Figure 6A and Table S7). We identified a series of small molecules that are likely to interact with LDHA^C163^ site. Among the top‐ranked candidates, surface plasmon resonance (SPR) validation revealed that Rutin exhibited the highest binding affinity to LDHA (Figure 6B,C and Figure S15A). Cellular thermal shift assay (CETSA) demonstrated that Rutin increased the thermal stability of LDHA, providing additional evidence for a direct molecular interaction (Figure S15C). Rutin, also known as vitamin P, is a flavonoid compound abundantly present in herbs, fruits, and vegetables [45]. It possesses favorable blood–brain barrier permeability and has demonstrated antitumor effects in various malignancies, including glioma, breast cancer, liver cancer, and colorectal cancer [46, 47, 48, 49]. However, the underlying mechanisms of its action have not been fully elucidated.

**FIGURE 6:**
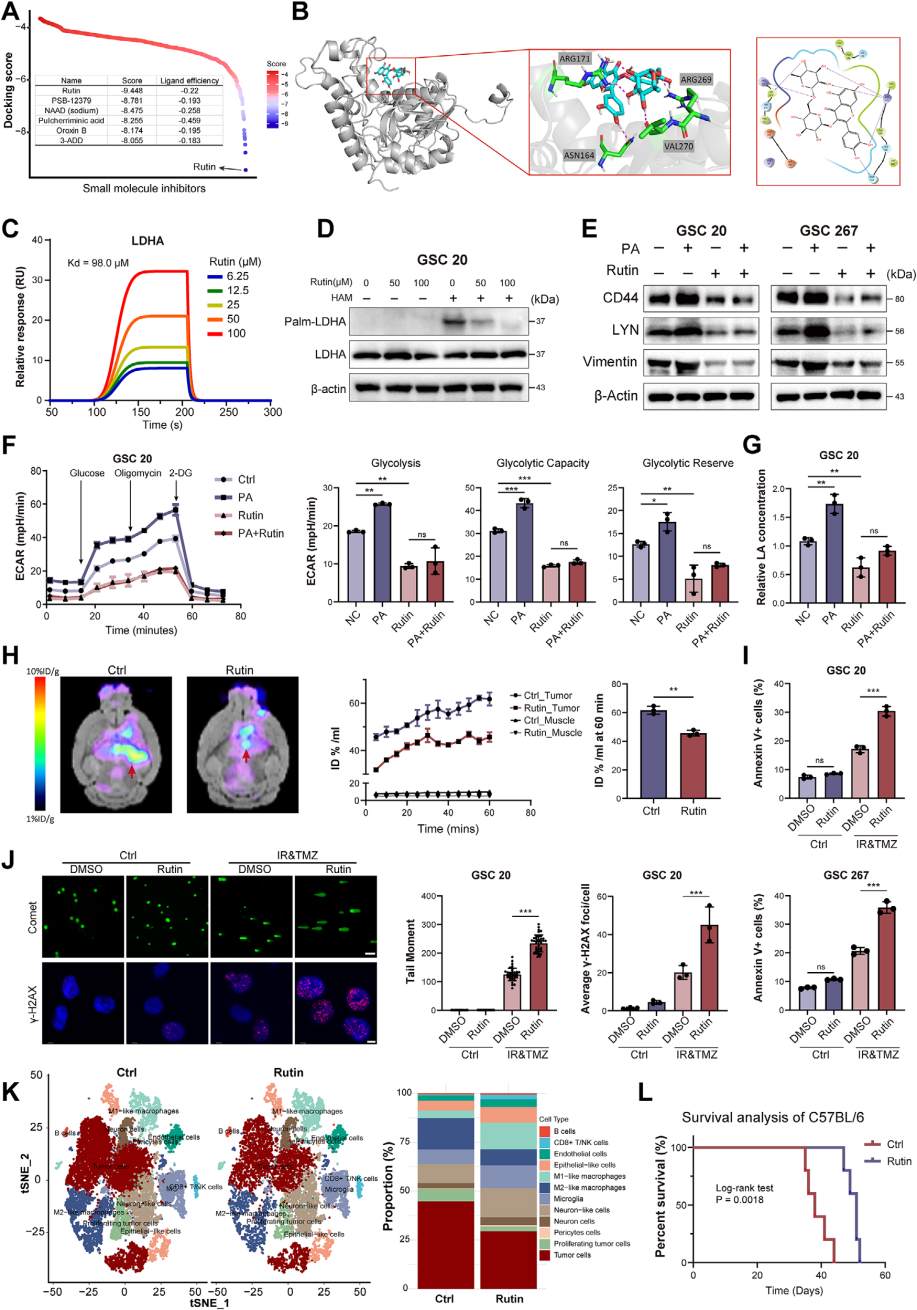
Screening of Rutin as an inhibitor of the LDHA palmitoylation site to enhance therapeutic efficacy in GSCs. (A) Virtual screening identifies rutin as a high‐affinity small molecule inhibitor targeting the palmitoylation site of LDHA (Cys163). (B) Molecular docking models illustrating the binding of Rutin to the LDHA active site and interaction with key amino acid residues. (C) Surface plasmon resonance (SPR) analysis showing the binding affinity of Rutin to LDHA. (D) ABE assay demonstrating that Rutin significantly inhibits LDHA palmitoylation. (E) Western blot analysis showing that Rutin blocks PA‐induced upregulation of MES markers in GSCs. (F) Seahorse analysis of ECAR in GSCs showing that Rutin abrogates PA‐induced increases in glycolysis, glycolytic capacity, and glycolytic reserve in GSC 20, n=3. (G) Measurement of lactate production in GSC 20 cells, n=3. (H) Representative micro‐PET CT images and quantification of ^18^F‐FDG uptake in intracranial tumor‐bearing mice treated with or without Rutin, n=3. (I) Apoptosis assays reveal that Rutin significantly restore chemoradiotherapy sensitivity in GSC 20 (upper) and GSC 267 (lower), n=3. (J) Representative images and quantification of alkaline comet assay (n=40; Scale bar, 50 µm) and γ‐H2AX IF (n=3; Scale bar, 5 µm) in GSC 20 cells. (K) tSNE visualization and proportion analysis of single‐cell transcriptomic data from glioma tissues. Bar plot shows the relative proportion of annotated cell types in Ctrl vs Rutin‐treated groups. (L) Kaplan‐Meier survival curve demonstrating prolonged survival of mice treated with Rutin compared to controls, n=5. Data are presented as mean ± sd. *p < 0.05, **p < 0.01, ***p < 0.001 by Student's t‐test or one‐way ANOVA as appropriate.

To clarify how Rutin inhibits LDHA palmitoylation, we examined whether it affects the interaction between LDHA and its palmitoyl‐transferase ZDHHC12. Co‐IP and PLA assays showed that Rutin did not substantially disrupt ZDHHC12‐LDHA binding or proximity (Figure S15E,F). However, ABE assays revealed that Rutin markedly reduced ZDHHC12‐mediated palmitoylation of LDHA^WT^, while LDHA^C163A^ displayed minimal palmitoylation and was largely insensitive to Rutin (Figure S15G). These findings indicate that Rutin suppresses LDHA palmitoylation mainly by binding to the Cys163‐centered palmitoylation site of LDHA, thereby blocking its modification, rather than by interfering with ZDHHC12‐LDHA interaction. Treatment with Rutin significantly inhibited LDHA palmitoylation, suppressed the MES subtype, and enhanced the therapy sensitivity of GSCs (Figure 6D and Figure S15B,D). Functionally, Rutin reversed the PA‐induced upregulation of MES markers in GSCs (Figure 6E). Extracellular flux analysis revealed that Rutin abrogated PA‐induced increases in glycolysis, glycolytic capacity, and glycolytic reserve, which was accompanied by a reduction in lactate production (Figure 6F,G and Figure S15H,I). Additionally, in vivo ^18^F‐FDG PET imaging revealed that Rutin‐treated mice exhibited significantly reduced glucose uptake in intracranial tumors, indicating suppression of tumor glycolytic activity (Figure 6H). Moreover, Rutin significantly elevated chemoradiotherapy‐induced apoptosis, DNA damage, as evidenced by increased comet tail moments and γH2AX foci formation (Figure 6I,J and Figure S15J,K), suggesting that Rutin enhances therapy sensitivity in GSCs by disrupting LDHA palmitoylation and glycolytic reprogramming.

Using immunocompetent mouse models, we investigated the role of Rutin in modulating the immunosuppressive TME in GBM. Rutin administration led to a marked reduction in tumor burden and a significant extension in survival (Figure 6L and Figure S16A,B). IF staining and Fc analysis further revealed decreased infiltration of Immunosuppressive tumor‐associated macrophages (Figure S16C–E). To further dissect the cellular landscape of the TME, we performed single‐cell transcriptomic profiling. tSNE visualization revealed a marked decrease in M2‐like immunosuppressive macrophages and proliferating tumor cells, along with an altered immune cell composition in Rutin‐treated tumors (Figure 6K). Collectively, these findings demonstrate that Rutin potently inhibits LDHA palmitoylation, thereby attenuating GSC chemoradiotherapy resistance and mitigating the immunosuppressive tumor microenvironment in GBM, highlighting its potential as a therapeutic agent.

### 2.10 Rutin Enhances the Therapeutic Efficacy of Chemoradiotherapy in GBM

To further assess the in vivo therapeutic potential of Rutin in combination with standard treatments, we established orthotopic glioma models and evaluated responses to radiotherapy and TMZ, with or without Rutin co‐administration (Figure 7A). Bioluminescence imaging revealed that the combination of IR, TMZ, and Rutin led to a significant reduction in tumor burden compared to IR/TMZ or Rutin alone (Figure 7B). Histological analysis further confirmed this observation, with H&E staining showing reduced tumor size in the combination group (Figure 7C), and γ‐H2AX IHC demonstrating enhanced DNA damage in the IR/TMZ/Rutin group (Figure 7D). Survival analysis demonstrated a substantial extension of overall survival in the triple‐treatment group compared to IR/TMZ or control groups, in both immunodeficient and immunocompetent mouse models (Figure 7E), supporting the synergistic effect of Rutin in enhancing therapeutic response. Tumor organoids serve as valuable models for drug discovery and therapeutic efficacy evaluation, providing a complementary platform to conventional animal models [50, 51]. Organoids treated with the triple combination therapy exhibited the highest levels of DNA damage, further validating the enhanced therapeutic efficacy of this regimen (Figure 7F). In addition to the conventional orthotopic model, we further established a postoperative recurrent GBM model by surgically resecting intracranial GSC‐luciferase tumors, followed by IR/TMZ and/or Rutin treatment (Figure 7G). IVIS imaging revealed that Rutin combined with IR/TMZ markedly delayed postoperative tumor recurrence and reduced tumor burden compared with either treatment alone (Figure 7H,I). Survival analysis further showed that the triple‐treatment group had the longest overall survival (Figure 7J). In conclusion, these findings systematically illustrated the proposed mechanism: MES GSCs, characterized by high fatty acid uptake and LDHA palmitoylation, exhibit elevated glycolysis and resistance to IR and TMZ. Rutin disrupts this axis by inhibiting LDHA palmitoylation, restoring therapy sensitivity and moderating the immunosuppressive TME. Collectively, these findings demonstrate that Rutin synergistically enhances the efficacy of radiotherapy and TMZ by targeting metabolic reprogramming and overcoming therapy resistance in MES‐type GBM (Figure 7K).

**FIGURE 7:**
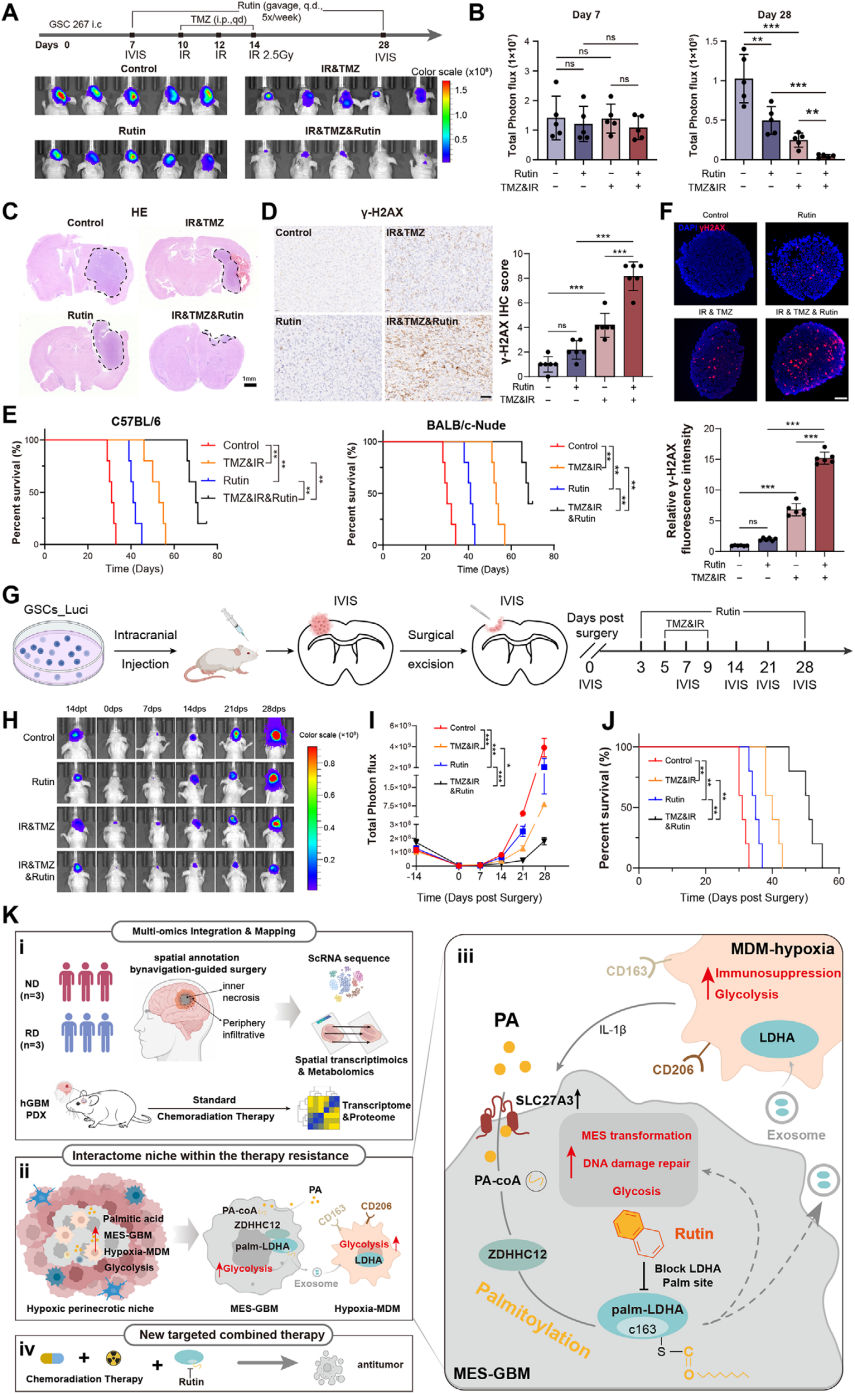
Rutin enhances the efficacy of chemoradiotherapy in vivo. (A) Schematic of in vivo experimental design and representative bioluminescence imaging of tumor‐bearing mice treated with indicated strategies. (B) Quantification of total photon flux at day 7 and day 28, showing significantly reduced tumor burden in the IR&TMZ plus Rutin group compared to other groups, n=5. (C) Representative H&E staining of mice brain sections at endpoint, demonstrating reduced tumor size in the triple combination therapy group. Scale bar, 1 mm. (D) IHC staining and scores for γ‐H2AX in brain tumor tissues, showing increased DNA damage in the triple combination therapy group, n=6. Scale bar, 50 µm. (E) Kaplan‐Meier survival analysis of both immunodeficient and immunocompetent tumor‐bearing mice demonstrated that the IR + TMZ + Rutin treatment group exhibited significantly improved survival compared to the control group, n=5. (F) Representative IF staining images and relative fluorescence intensity of γ‐H2AX in GBM organoids, n=6. Scale bars, 100 µm. (G) Schematic of the experimental workflow. (H) Representative bioluminescence images of intracranial tumors at indicated time points after surgery. (I) Quantification of total photon flux showing that Rutin combined with TMZ/IR markedly suppressed postoperative tumor regrowth, n=5. (J) Kaplan‐Meier survival analysis showing prolonged survival in mice receiving the triple combination treatment, n=5. (K) A graphical summary illustrating the overall analytical framework and the mechanisms by which PA promotes chemoradiotherapy resistance in GBM through defined molecular and metabolic pathways. Data are presented as mean ± sd. *p < 0.05, **p < 0.01, ***p < 0.001 by Student's *t*‐test or one‐way ANOVA as appropriate.

## 3 Discussion

Surgical resection remains the cornerstone of GBM treatment. However, the highly infiltrative nature of GBM, combined with the need to preserve functional brain regions, often precludes complete tumor removal [52]. As a result, postoperative adjuvant therapies are essential for eliminating residual tumor cells, and understanding resistance to such therapies is particularly critical in incompletely resectable malignancies like GBM. If surgical resection represents a coordinated assault, postoperative chemoradiotherapy serves to eliminate residual stragglers. However, a subset of tumor cells either intrinsically resists therapy or acquires resistance through treatment‐induced selective pressure or mutagenesis. These resistant populations ultimately drive GBM recurrence and treatment failure. To better model this process, we established orthotopic intracranial GBM patient‐derived xenograft (PDX) models using tumor tissues from GBM patients and administered a standard chemoradiotherapy regimen. Multi‐omics analysis of GBM cells that had acquired therapy resistance revealed a selective enrichment of the MES subtype, consistent with previous reports implicating MES GSCs in driving treatment resistance and postoperative recurrence of GBM [53]. Through integrated data analysis and experimental validation, we found that both therapy resistant cells and MES GSCs exhibited markedly elevated fatty acid transport activity. This metabolic feature suggests a dependency on exogenous lipid uptake to support survival under therapeutic stress, prompting us to investigate the downstream consequences of fatty acid–driven metabolic reprogramming in MES GSCs.

Building on this link between fatty acid metabolism and therapy resistance, we further investigated the downstream molecular mechanisms by which elevated lipid uptake promotes chemoradiotherapy resistance in GBM. A metabolite screen of fatty acid derivatives identified PA as a key molecule capable of attenuating the cytotoxic effects of therapy on GSCs. Additionally, multi‐omics analysis, through mapping metabolites to therapy‐resistant regions, further validated that PA drives MES GBM progression and therapeutic resistance. PA is the most abundant dietary saturated fatty acid, commonly derived from animal fat and palm oil, and serves not only as a critical component of membrane phospholipids and an energy substrate via β‐oxidation, but also as a signaling molecule implicated in inflammation, immune modulation, and cancer progression. Recent study has shown that mice subjected to a short‐term palm oil–rich diet, as well as tumor cells briefly exposed to PA in vitro, exhibited sustained high metastatic potential [54]. Another study revealed that intra‐tumoral accumulation of PA drives extracellular matrix remodeling by promoting the transition of normal fibroblasts into matrix‐associated cancer‐associated fibroblasts via tumor cell–derived cytokine signaling, thereby enhancing tumor progression and tissue stiffening [55]. While PA is widely utilized by tumor cells as a key metabolic substrate, it has also been associated with lipid peroxidation and ferroptosis under certain conditions. In our study, however, exogenous PA pre‐administration promoted the survival and therapy resistance of GSCs, suggesting that its intracellular fate may be context‐dependent. Rather than contributing to oxidative lipid stress, PA may be selectively directed toward alternative pathways, such as protein palmitoylation, that support tumor adaptation during therapeutic challenge. Consistent with this hypothesis, our experiments demonstrated that PA enhances therapy resistance in GSCs primarily through protein S‐palmitoylation.

The study of protein palmitoylation has long been limited by the absence of a consensus substrate motif and the reliance on low‐sensitivity, labor‐intensive methods such as radioactive [^3^H]‐palmitate labeling. The development of the acyl‐biotin exchange (ABE) assay has markedly improved the efficiency and sensitivity of palmitoylation detection [56, 57, 58]. Using the ABE assay on GBM tissue samples and GSCs, we identified a significant enrichment of palmitoylated proteins within the glycolytic pathway, suggesting a strong association between PA‐mediated palmitoylation and metabolic reprogramming in GBM. Further analysis revealed ZDHHC12 as a key palmitoyl‐transferase responsible for regulating GSC glycolysis and resistance to chemoradiotherapy. ZDHHC12 has been reported to palmitoylate NLRP3, promoting its degradation via the chaperone‐mediated autophagy pathway [59, 60, 61]. Additionally, ZDHHC12‐mediated palmitoylation of MAVS has been shown to facilitate its activation in antiviral innate immune responses [62]. However, the functional role of ZDHHC12 in cancer, particularly in glioma, remains largely unexplored. In this study, we demonstrate that ZDHHC12 catalyzes the palmitoylation of LDHA, thereby promoting glycolytic reprogramming and contributing to therapy resistance in GBM. Palmitoylation enhances the hydrophobicity of substrate proteins, thereby influencing their subcellular localization, stability, enzyme activity, membrane association, and protein–protein interactions. We systematically investigated each of the above functional roles and ultimately found that ZDHHC12‐mediated palmitoylation primarily regulates the enzymatic activity of LDHA and facilitates its translocation into exosomes. The former promotes maintenance of the MES subtype of GSCs and attenuates the cytotoxic effects of chemoradiotherapy by enhancing glycolytic metabolism, while the latter contributes to the establishment of an immunosuppressive TME by modulating TAM in GBM. Recent studies have revealed that TAMs secrete extracellular vesicles enriched in LDHA, which enhance glycolysis, proliferation, and survival of GBM cells [63]. In contrast, we identified that palmitoylated LDHA is also secreted by GSCs via exosomes, where it contributes to the induction of an immunosuppressive phenotype in infiltrating macrophages. Notably, targeting LDHA palmitoylation significantly disrupted the crosstalk between TAMs and GBM cells. Therefore, our study provides important insights into the regulatory mechanisms and functional roles of LDHA in GBM, while also highlighting the therapeutic potential of targeting its palmitoylation.

Importantly, our study reveals LDHA palmitoylation as a previously unrecognized mechanism linking fatty acid uptake to metabolic reprogramming and therapeutic resistance in GBM. Rather than serving merely as an energetic substrate or membrane component, PA promotes ZDHHC12‐mediated LDHA palmitoylation, thereby enhancing LDHA enzymatic activity, sustaining the MES phenotype, and facilitating exosomal LDHA release to remodel the immunosuppressive TME. These findings extend the functional significance of LDHA beyond canonical glycolysis and identify LDHA palmitoylation as a metabolic–immune regulatory node in GBM recurrence. Therapeutically, targeting this modification may selectively disrupt the therapy‐resistant LDHA pool and provide a promising strategy to enhance the efficacy of standard chemoradiotherapy.

Given the pathological significance of LDHA palmitoylation in GBM, we sought to identify pharmacological agents capable of disrupting this modification. Previous studies have shown that competitive inhibitors, such as peptides containing palmitoylation motifs, can block the palmitoylation of target proteins [40, 41, 64]. However, the clinical translation of such short peptides remains challenging. In our study, we identified cysteine 163 (C163) as the critical site for LDHA palmitoylation. Based on this finding, we performed structure‐based screening and identified Rutin as a small molecule capable of occupying the C163 site, thereby inhibiting LDHA palmitoylation and enzymatic activity. Rutin is a flavonoid compound with favorable blood–brain barrier permeability, and has been reported to exert antioxidant, anti‐inflammatory, anti‐tumor, and anti‐aging effects [65, 66, 67, 68, 69]. Its multifunctional properties and bioavailability make it a promising candidate for therapeutic applications in central nervous system disorders. Recent studies developed a hybrid layered composite nanofiber mesh co‐loaded with TMZ and Rutin, which exhibited a pronounced antitumor effect [49, 70]. However, the precise molecular mechanism underlying Rutin's activity remains unclear. Our findings demonstrate that Rutin blocks LDHA palmitoylation and inhibits its enzymatic function, thereby enhancing tumor cell sensitivity to chemoradiotherapy and exerting potent antitumor effects in combination therapy in vitro and in vivo.

In conclusion, this study demonstrates that PA accumulation is a defining feature of the hypoxic pseudopalisading necrosis regions in GBM, where it drives MES subtype maintenance and therapeutic resistance. Mechanistically, MES GSCs preferentially uptake PA, which promotes LDHA palmitoylation via the ZDHHC12. Palmitoylated LDHA operates via two distinct mechanisms: by enhancing enzymatic activity to promote glycolytic reprogramming, and by being secreted in exosomes to regulate TAM. Furthermore, targeting the palmitoylation site of LDHA (Cys163) with the small molecule Rutin significantly reduced LDHA palmitoylation and increased the chemoradiotherapy sensitivity of MES GSCs. These findings uncover a previously unrecognized regulatory mechanism of LDHA palmitoylation in GBM and highlight a potential therapeutic strategy for overcoming treatment resistance.

## 4 Experimental Section

### 4.1 Human Samples

Human glioma tissue samples were collected from patients undergoing surgical resection at Qilu Hospital of Shandong University. The use of human samples and associated clinical data was approved by the Medical Ethics Committee of Qilu Hospital, Shandong University. All participants provided written informed consent, and the study was approved by the Ethics Committee on Scientific Research of Shandong University Qilu Hospital (approval number: KYLL‐2023(ZM)‐657). Tumor tissues were used for histological evaluation, IHC, and molecular analyses, including RNA sequencing and other downstream assays.

### 4.2 Cell Lines and Cell Culture

Patient‐derived PN‐GSCs (GSC 8‐11, GSC 11), MES‐GSCs (GSC 20, GSC 267, GSC 28), and neural progenitor cells (NPCs) were generously provided by Dr. Frederick F. Lang and Dr. Krishna P.L. Bhat (MD Anderson Cancer Center, University of Texas). GSC subtypes were classified based on the genetic signatures described by Phillips and Verhaak, which are widely accepted and used for GBM subtype study. GSCs and NPCs were dissociated into single cells using Accutase (A6964, Sigma‐Aldrich) and cultured in DMEM/F12 (A4192001, Gibco) supplemented with B‐27 (17504044, Gibco), 20 ng/mL recombinant human EGF (236‐EG, R&D Systems), and 20 ng/mL bFGF (3718‐FB, R&D Systems). The human GBM cell lines U87MG (RRID: CVCL_0022) and U251 (RRID: CVCL_0021) (Chinese Academy of Sciences Cell Bank) and mouse GBM cell line GL261 (RRID: CVCL_Y003, ATCC) were cultured in DMEM (D6429, Sigma) supplemented with 10% FBS (A5670701, Thermo Fisher Scientific). The THP1 cell lines (RRID: CVCL_0006, Chinese Academy of Sciences Cell Bank) were cultured in RPMI1640 (R8758, Gibco) with 10% FBS. All cells were maintained at 37°C in a humidified incubator with 5% CO_2_ and 5% O_2_.

### 4.3 Single Cell Data Preprocess

Raw FASTQ reads were aligned to the GRCh38/hg38 reference genome with Cell Ranger v3.0 (10× Genomics). A Cell Ranger–compatible reference was constructed following the 10× Genomics guidelines. The Cell Ranger pipeline then carried out read alignment and quality filtering, demultiplexed cellular barcodes, and quantified UMIs. After mapping, digital gene expression (DGE) matrices were produced for each individual sample as well as for the combined dataset. Sample‐level DGE outputs were integrated using Cell Ranger's aggr function, with libraries depth‐normalized by balancing effective read counts across batches. This yielded a unified expression matrix containing all cells and their transcript counts across genes.

### 4.4 TSNE Visualization and Determination of the Major Cell Types

After quality control, downstream expression analysis and cell‐type assignment were performed in R using Seurat. Because the input counts had already been processed/normalized upstream, the data were imported into Seurat without additional normalization, scaling, or centering steps. Per‐cell metadata (including spatial/location information) were incorporated alongside the expression matrix. Highly variable genes were then selected and used for principal component analysis (PCA). The PC loadings and associated genes were evaluated to choose the number of components retained for subsequent analyses. Cells were visualized in two dimensions using t‐SNE based on the top 20 principal components. Cluster‐specific differentially expressed genes were identified with Seurat's FindAllMarkers, requiring detection in at least 10% of cells, a log fold‐change > 0.25, and statistical significance (adjusted *p* < 0.05; Wilcoxon rank‐sum test). Finally, clusters were assigned to known cell types by comparing their marker profiles with established canonical markers.

### 4.5 Pseudotime Analysis

Single‐cell trajectories were inferred using Monocle 2 on the cell‐by‐gene expression matrix. Genes showing substantial variation across cells (or differential expression across states) were selected as ordering features to reconstruct the dynamic trajectory and arrange cells along pseudotime. Briefly, gene‐level transcript abundance was quantified for each cell, and genes were prioritized by their relationship between coefficient of variation and mean expression. For each cell type, the top 20 ranked genes were retained as trajectory features. In parallel, RNA velocity estimates were computed and overlaid onto the UMAP embedding generated in Seurat to visualize directional transitions among cell states.

### 4.6 Functional Analysis of Differential Genes

Differential gene expression across cell types was assessed in Seurat using the Wilcoxon rank‐sum test. To estimate pathway activity at single‐cell resolution, gene set variation analysis (GSVA) was performed with default parameters using the GSVA package. Gene set enrichment analysis (GSEA) was conducted with GSEA software based on curated gene sets from the Molecular Signatures Database (MSigDB).

### 4.7 Spatial Transcriptomics Data Processing

Briefly, data were normalized with SCTransform, followed by dimensionality reduction with PCA and UMAP. Clustering was performed using the first 20 principal components at the default resolution. Spatial expression patterns were visualized using SpatialFeaturePlot, and signature scores for MES‐like malignant cells and monocyte‐derived macrophages (MDMs) were computed using AddModuleScore.

### 4.8 Spatial Metabolomics

Sample preparation: Tissues were stored at −80°C prior to analysis. Tissues were placed at −20°C 30 min before use. Before sectioning, fix the frozen tissue samples onto the tray with water. The tissues were sectioned at 10 µm thickness using a Leica CM1950 cryostat (Leica Microsystems GmbH, Wetzlar, Germany) at −20°C. Subsequently, the cut tissue sections were attached to the adhesive microscope slides, and the slides with tissue sections were dried in a vacuum desiccator for 30 min. AFADESI Parameter Settings: Place the adhesive microscope slides with tissue sections on the AFADESI mobile platform. Determine the scanning area according to the size of the tissue, and the scanning is carried out in a line‐by‐line scanning manner. The composition of the spraying solvent is acetonitrile: water = 90:10 (V/V), and the flow rate of the spraying solvent is 3 µL/min. The pressure of the spraying gas is 0.65 Mpa. The spraying voltage is 4500 V in the positive mode and −4000 V in the negative mode. Mass spectrometry imaging: The metabolites in the samples were imaged using the AFADESI imaging platform. The mass spectrometry scanning range was 100–1300 Da. The Massimager software was used to process the data and extract the peaks. The substances of the mass spectrometry peaks are identified by matching the primary molecular weights with those in the database.

### 4.9 Antibodies and Reagents

The following antibodies were generated by Cell Signaling: anti‐CD44 (37259, RRID: AB_2750879); anti‐LYN (2796, RRID: AB_2138391); anti‐γH2AX (Ser139) (9718, RRID: AB_2118009); anti‐PKM2 (4053, RRID: AB_1904096); anti‐Flag (D6W5B) (14793, RRID: AB_2572291); anti‐β‐Tubulin (2128, RRID: AB_823664); anti‐CD63 (52090, RRID: AB_2924771); anti‐Cd163 (93498, RRID: AB_2800204); anti‐Cd206 (24595, RRID: AB_2892682) and anti‐Caspase3 (9662, RRID: AB_331439). The following antibodies were generated by Proteintech: anti‐LDHA (19987‐1‐AP, RRID: AB_10646429); anti‐Vimentin (10366‐1‐AP, RRID: AB_2273020); anti‐β‐Actin (66009‐1‐Ig, RRID: AB_2687938) and anti‐SLC27A3 (12943‐1‐AP, RRID: AB_2286200). The anti‐ZDHHC12 (PA5‐113144) was obtained from Invitrogen. For western blots, antibodies were diluted 1:1000. For immunofluorescence, antibodies were diluted 1:200. For IHC, antibodies were diluted 1:100. Rutin (HY‐N0148), Etomoxir (HY‐50202) and TMZ (HY‐17364) were purchased from MedChemExpress. Palmitic acid (P0500) and 2‐BP (21604) were purchased from Sigma‐Aldrich. BODIPY FL C16 (D3821) was purchased from Invitrogen.

### 4.10 Fatty Acid Uptake Test

For fluorescence imaging of fatty acid uptake, 2 × 10^4^ indicated GSC cells were seeded into pre‐coated 8‐well chamber slides (ibidi) and incubated with 1 µM BODIPY FL C16 in culture medium for 12 h. Cells were then washed three times with PBS, and fluorescent fatty acid signals were visualized using a Leica TCS SP8 confocal microscope.

For the flow cytometric analysis of fatty acid uptake, 2 × 10^5^ GSC cells of a defined subtype were seeded into 12‐well plates and treated with BODIPY FL C16 (final concentration: 25 µM) for 30, or 60 min. After three washes with PBS, fatty acid uptake was quantified by flow cytometry (Agilent Technologies).

Fatty acid uptake was also assessed using the Fatty Acid Uptake Kit (MAK156, Millipore Sigma) following the manufacturer's instructions. GSC cells were seeded into 96‐well plates at a density of 1 × 10^5^ cells per well. The culture medium was replaced with 100 µL of Fatty Acid Dye Loading Solution, and fluorescence intensity (λ_ex_ = 485 nm, λ_em_ = 515 nm) was measured after 1, 10, and 30 min of incubation using a BioTek Synergy plate reader.

### 4.11 Xenograft Models

All animal studies were performed in accordance with Institutional Animal Care and Use Committee (IACUC) guidelines and were approved by the IACUC of the Qilu Hospital of Shandong University (Approval No. DWLL‐202400062). Male C57BL/6 (RRID: MGI: 2159769) or BALB/c nude (RRID: MGI: 2161072) mice (aged 4–6 weeks), were obtained from GemPharmatech Co., Ltd. (Nanjing, Jiangsu, China) and housed at the Neurosurgery Laboratory of Qilu Hospital of Shandong University (Jinan, Shandong, China).

To evaluate the efficacy of standard in vivo chemoradiotherapy in eliminating therapy‐sensitized GBM progenitor cells, PDX models were established in BALB/c nude mice. 7 days after tumor implantation, mice were randomly assigned to either a control group or an IR+TMZ group. Mice in the IR+TMZ group received irradiation at 2 Gy per fraction, once daily for five consecutive days, with synchronous TMZ administration at 25 mg/kg daily via intraperitoneal injection. At 28 days post‐tumor implantation, GBM tissues were harvested for in vitro culture or subjected to deep sequencing.

To investigate palmitate diet–induced chemoradiotherapy resistance in vivo, BALB/c nude mice were randomized into a high‐palmitic acid diet group (supplemented with palm oil, TD150067, Envigo) and a normal diet group prior to intracranial tumor implantation. Luciferase‐expressing GSC 267 cells (5 × 10^5^ per mouse) were implanted intracranially. 7 days after tumor seeding, mice were further randomized into control and IR+TMZ groups. Mice in the IR+TMZ group received irradiation at 2.5 Gy per fraction every other day for a total dose of 7.5 Gy, combined with daily TMZ treatment (25 mg/kg, i.p.) from day 10 to day 14. Tumor progression was monitored using the IVIS Spectrum in vivo imaging system (PerkinElmer Inc., Waltham, MA, USA) on days 7 and 28 post‐implantation.

In animal experiments involving Rutin combined with standardized treatment, mice were acclimated and then orthotopically implanted with GSC 267 on Day 0. Rutin treatment (100 mg/kg, oral gavage, once daily) started on Day 7 post‐implantation and continued until Day 28. Mice in the IR&TMZ and IR&TMZ&Rutin groups received TMZ (25 mg/kg, i.p., daily) from Day 10 to 14 and focal cranial irradiation (2.5 Gy per session) on Days 10, 12, and 14. Bioluminescent imaging (IVIS) was performed on Days 7 and 28 to monitor tumor burden.

All mice were housed in a specific pathogen‐free (SPF) barrier facility with individually ventilated cages. Anesthesia was administered using isoflurane (#R510‐22‐10, RWD Life Science), with induction in a 2.0%–2.5% isoflurane/oxygen mixture and maintenance in 1.5%–1.8% isoflurane/oxygen. Moribund animals were identified based on the following criteria: (1) difficulty in breathing, feeding, or drinking; or (2) ≥15% body weight loss within 4 days. To minimize suffering, animals were exposed to high‐concentration CO_2_ (30%) for at least 3 min until complete cessation of respiration was observed.

### 4.12 Isolation and Culture of Primary GBM Cells

Surgically resected GBM specimens from patients at Qilu Hospital, Shandong University, China, as well as tumor samples extracted from the brains of mice, were processed similarly. Briefly, after washing with precooled DMEM containing streptomycin and penicillin, the tumor tissues were gently minced and digested using the tissue enzyme‐lysis kit (DHTEH‐2505. RWD Life Science) following the instructions. Tumor cells were then filtered through a 200‐mesh filter to obtain a single‐cell suspension, and then erythrocytes were removed using erythrocyte lysate (WL1000, R&D Systems). Finally, the primary GBM cells were cultured in serum‐free medium (DMEM/F12, 2% B‐27, 20 ng/mL EGF, 20 ng/mL bFGF).

### 4.13 Chemoradiotherapy Treatment

Radiation treatment for both in vitro and in vivo experiments was performed using an X‐ray irradiator (X‐Rad225 OptiMAX, PXi). And TMZ (HY‐17364) were purchased from MedChemExpress. For in vitro assays, tumor cells were exposed to ionizing radiation (6 Gy) and TMZ (200 µM), and subsequent functional analyses were carried out 48 h after chemoradiotherapy. For in vivo studies, mice received irradiation at the indicated dose and were treated with TMZ (25 mg/kg) between days 10 and 14 following tumor cell implantation. A detailed schematic of the treatment regimen is shown in the figure.

### 4.14 RNA‐seq and Data Analysis

1. Sample Preparation and Library Construction. Total RNA was extracted and assessed for integrity and purity according to standard protocols. Messenger RNA (mRNA) was enriched using poly‐T oligo‐attached magnetic beads. For non‐strand‐specific libraries, cDNA was synthesized using random hexamer primers for both strands. For strand‐specific libraries, the second strand was synthesized using dUTP to maintain directionality. Libraries were prepared through end repair, A‐tailing, adaptor ligation, size selection, and PCR amplification. Library quality and concentration were evaluated using Qubit, real‐time PCR, and Bioanalyzer. 2. Sequencing. Qualified libraries were pooled based on concentration and expected data yield, and subjected to Illumina sequencing. Sequencing was performed using the Sequencing by Synthesis (SBS) strategy, where fluorescently labeled nucleotides were incorporated and imaged in real‐time to generate raw reads. 3. Bioinformatics Analysis. Quality Control: Raw reads were processed using fastp to remove adaptors, poly‐N sequences, and low‐quality reads. Quality metrics including Q20, Q30, and GC content were calculated. Read Mapping: Clean reads were aligned to the reference genome using HISAT2 (v2.2.1, RRID: SCR_015530) with the corresponding gene annotation file to ensure splice‐aware alignment. Expression Quantification: Gene‐level counts were obtained using featureCounts (v2.0.6). Gene expression was normalized as FPKM (Fragments Per Kilobase of transcript per Million mapped reads). Differential Expression Analysis: For datasets with biological replicates, differential expression analysis was performed using DESeq2 (RRID: SCR_000154, v1.42.0) based on a negative binomial distribution. For datasets without replicates, edgeR (v4.0.16) was used with scaling normalization. Differentially expressed genes (DEGs) were identified using a threshold of padj ≤ 0.05 and | log_2_(fold change) | ≥ 1.

### 4.15 Public Data Collection and Analysis

Transcriptomic data and corresponding clinical information for TCGA GBM were obtained from the GDC Data Portal (https://portal.gdc.cancer.gov/). RNA‐seq data and clinical annotations for CGGA GBM samples were downloaded from the CGGA database (http://www.cgga.org.cn/). Transcriptome profiles for the Rembrandt (GSE108476) and Gravendeel (GSE16011) GBM cohorts were retrieved from the GEO database (https://www.ncbi.nlm.nih.gov/geo/). Single‐cell RNA sequencing data were obtained from GSE131928.

The fatty acid transport activity gene signature (GO:0015908) was obtained from the GSEA website (https://www.gsea‐msigdb.org/gsea/index.jsp, RRID: SCR_016863). Single‐cell RNA‐seq data were analyzed using the R package Seurat (v4.1.0), and t‐SNE was applied for visualization of distinct cell clusters. The R package irGSEA was used to calculate and visualize enrichment scores for the FAT signature using the “UCell” method. For GSEA analysis of TCGA bulk RNA‐seq data, gene signatures were also downloaded from the GSEA website, and enrichment analysis was performed using GSEA software (v4.1.0).

### 4.16 Plasmid, siRNA and Cell Transfection

All shRNA and overexpression plasmids were purchased from GenePharma (Shanghai, China). Selected shRNA sequences were cloned into the pLKO.1‐puro lentiviral backbone. Recombinant lentiviral particles were generated by co‐transfecting 293T cells (RRID: CVCL_0063) with 5 µg of the target plasmid, 3.25 µg of psPAX3, and 1.75 µg of pMD2.G packaging plasmids using linear polyethylenimine (PEI; 23966, Polysciences). Lentiviral supernatants were harvested 48 h after transfection, filtered through a 0.45 µm membrane (Corning), and used to infect target cells in the presence of 8 µg/mL polybrene. After 24 h of infection, cells were selected with 2 µg/mL puromycin (P8833, Sigma‐Aldrich) to establish stable lines. For transient gene silencing or overexpression, cells were transfected with siRNAs or plasmids using the Lipofectamine 3000 reagent (L3000015, Invitrogen), following the manufacturer's protocol. The RNA‐interfering sequences used in this study are listed in Table S8.

### 4.17 HR Reporter Assays

To evaluate the effect of PA on homologous recombination (HR) repair of DNA double‐strand breaks (DSBs), GSC 267 cells stably integrated with DR‐GFP plasmid (RRID: Addgene_26475). Following 48 h of PA treatment, approximately 5 × 10^5^ GSC 267 DR‐GFP cells were transfected with 1 µg of the pCMV‐I‐SceI using Lipofectamine 3000 Transfection Kit (Invitrogen). GFP fluorescence was measured 3 days post‐transfection using a flow cytometer (Agilent Technologies). HR repair efficiency was determined by calculating the percentage of GFP‐positive cells among the total number of analyzed cells.

### 4.18 Western Blotting (WB), Immunohistochemistry, and Immunofluorescence (IF)

For Western blotting, GSCs or GBM tissues were lysed in RIPA buffer (9806, Cell Signaling Technology) supplemented with 1% protease and phosphatase inhibitors (IKM1010, Solarbio). Protein lysates were separated via SDS‐PAGE and transferred to PVDF membranes. Membranes were incubated with primary antibodies overnight at 4°C, followed by incubation with appropriate secondary antibodies at room temperature for 1 h. Protein signals were detected using an Odyssey fluorescence scanner (ChemiDoc XRS+, Bio‐Rad, USA).

For IHC, tissue sections from GSC xenograft‐bearing mice were stained with anti‐γH2AX or anti‐ZDHHC12 primary antibodies. Images were captured using a light microscope and quantitatively evaluated based on the German IHC scoring system.

For IF, GSCs were seeded and fixed in µ‐slide 8‐well plates (ibidi). Cells were permeabilized with 0.3% Triton X‐100 and blocked with 5% goat serum. After overnight incubation with primary antibodies at 4°C, cells were stained with fluorescent secondary antibodies and DAPI (D9542, Sigma‐Aldrich) for 1 h and 15 min, respectively. Cells were washed three times with PBS after each step. Representative images were acquired using a Leica SP8 confocal microscope.

### 4.19 Apoptosis Assay

Tumor cells from different experimental groups were harvested and enzymatically dissociated into single‐cell suspensions using Accutase, followed by gentle washing with PBS to avoid mechanical damage caused by excessive pipetting. Apoptosis was assessed using the Annexin V‐FITC Apoptosis Detection Kit (556547, BD Biosciences), following the manufacturer's protocol. Briefly, cells were resuspended in 100 µL of binding buffer, and 2.5 µL of Annexin V‐FITC and 5 µL of PI were added. The mixture was incubated for 15 min at room temperature in the dark. Following incubation, cells were washed once with PBS to terminate the staining reaction and resuspended in 300 µL of flow cytometry buffer. Samples were analyzed using a flow cytometer (Agilent Technologies), and the percentages of apoptotic cells were quantified.

### 4.20 Screening for Fatty Acid Metabolites Associated With Therapy Resistance

GSC 267 cells were treated with a human endogenous fatty acid compound library (MedChemExpress) containing about 200 metabolic molecules at concentrations of 100 µM. Following treatment, cells were divided into two groups: a control group and chemoradiotherapy group (6 Gy + 200 µM TMZ). 48 h post‐treatment, cell viability was assessed using a standard viability assay. The relative cell survival was determined by calculating the ratio of cell viability in treatment group versus control cells for each fatty acid treatment: GSC _(FA+T)_ / GSC _(FA)_.

### 4.21 Alkaline Comet Assay

The comet assay was conducted using the Single Cell Gel Electrophoresis Assay Kit (Catalog #4250‐050‐K, R&D Systems,) in accordance with the manufacturer's instructions. GSCs from different groups were resuspended in PBS at a concentration of 1 × 10^5^ cells/mL. A 10 µL aliquot of the cell suspension was mixed with 100 µL of 0.5% low melting point agarose (LMA) dissolved in calcium‐ and magnesium‐free phosphate‐buffered saline (PBS) at 37°C. Then, 50 µL of the mixture was dropped onto a microscope slide and allowed to solidify at 4°C for 10 min. The slides were then immersed in freshly prepared lysis buffer and incubated overnight at 4°C. After lysis, the slides were washed twice with PBS. An alkaline unwinding buffer was prepared using 0.4 g NaOH, 250 µL of 200 mM EDTA, and double‐distilled water to a final volume of 50 mL, then cooled to room temperature. The slides were incubated in this buffer for 20 min at room temperature in the dark to unwind the DNA and expose single‐strand breaks, facilitating DNA migration during electrophoresis. The electrophoresis buffer was prepared by mixing 8 g NaOH and 2 mL of 500 mM EDTA in 1 L of double‐distilled water and cooled to 4°C. Slides were placed in a horizontal electrophoresis tank filled with 850 mL of the cold alkaline buffer and subjected to electrophoresis at 25 V / 300 mA for 30 min. After electrophoresis, the slides were removed, and excess buffer was gently blotted with filter paper. Slides were washed twice with PBS and immersed in 70% ethanol for 5 min, followed by a final PBS rinse. Slides were then air‐dried in a 37°C oven for 10–15 min. Each slide was stained with 50 µL of SYBR Green solution in the dark for 30 min. After a PBS rinse, slides were observed and imaged under a fluorescence microscope (Nikon eclipse ti2). The tail moment was calculated using the OPENCOMET plugin for Fiji (RRID: SCR_002285).

### 4.22 Acyl‐Biotin Exchange Assay

The ABE assay was performed to detect protein palmitoylation as previously described. Lysis buffer was freshly prepared by mixing 10 mL of base lysis buffer with 100 µL PMSF and 100 µL protease/phosphatase inhibitor cocktail (IKM1010, Solarbio). N‐ethylmaleimide (NEM, 04260, Sigma) was dissolved in anhydrous ethanol to prepare a 2 M stock solution. For each sample, 20 mL of lysis buffer was mixed with 0.5 mL of 2 M NEM to block free thiol groups. Cells were lysed in 500 µL of the above buffer and incubated on ice for 10 min. Protein concentrations were quantified using the BCA assay to ensure equal protein input across groups (minimum of 500 µg per sample). Samples were incubated with 5 µg of primary antibody overnight at 4°C with gentle agitation. The next day, 50 µL of Protein A/G magnetic beads (88802, Thermo Fisher Scientific) were added to each sample and incubated for 1 h at 4°C with rotation. Tubes were gently inverted or vortexed briefly to mix, and magnetic separation was used to collect beads. The beads were then resuspended in 600 µL of lysis buffer supplemented with 10 mM NEM. From this suspension, 200 µL of bead‐bound lysate was transferred to a new microcentrifuge tube and labeled as the hydroxylamine (HAM, 159417, Sigma) (–) group; 300 µL of lysis buffer containing 10 mM NEM was added to this tube. The remaining 400 µL of bead‐bound lysate was mixed with 100 µL of the same buffer and labeled as the HAM (+) group. Both groups were incubated on ice for 10 min. Beads were washed once with 0.5 mL of buffer. HAM elution buffer (1 M HAM, freshly prepared in lysis buffer) was added to the HAM (+) samples, while lysis buffer alone was added to the HAM (–) controls. Samples were incubated on a rotator at room temperature for 45 min. Biotin‐BMCC (21900, Thermo Fisher Scientific) was prepared by dissolving 2.1 mg in 0.5 mL of DMSO to yield an 8 mM stock solution. Beads were washed again with lysis buffer using magnetic separation, then incubated with 0.5 mL of biotin‐BMCC working solution (5 µM in lysis buffer) at 4°C for 45 min with gentle mixing. Biotinylated proteins were then incubated with streptavidin–agarose beads (SA10004, Thermo Fisher Scientific) to capture palmitoylated proteins via the strong biotin–streptavidin interaction. Bound proteins were subsequently eluted and purified for downstream analysis such as immunoblotting.

### 4.23 Micro‐PET/CT Imaging of Mice

The indicated GSC 267 cells (5 × 10^5^, 10 uL) were injected into the brains of BALB/c nude mice (4–5 weeks old). PET imaging was performed 14 days after tumor cells inoculation. The tumor‐bearing mice were deprived of food and water for 8–10 h before micro‐PET/CT imaging. The mice were subsequently injected with approximately 300 µCi of ^1^ ^8^F‐FDG via the tail vein, followed by dynamic PET imaging acquisition every 5 min using a PET/CT/FMT system (MadicLab PSA094). All animals were anesthetized with isoflurane (2%–3% in 1 L/min oxygen) and placed on a heating pad to maintain body temperature throughout the imaging procedure. Respiratory rate and signs of distress were continuously monitored. Data acquisition began immediately following intravenous tracer injection. Micro‐PET/CT images were analyzed using PMOD software (version 4.0; PMOD Technologies Ltd., Zurich, Switzerland). Each PET image was manually co‐registered to the Mirrione mouse brain template using rigid body transformation, and the resulting parameters were applied to the corresponding PET datasets. Inveon Research Workplace software was used to quantify the percentage of injected dose per gram (%ID/g) and the SUV. The SUVmax data were plotted and analysed.

### 4.24 Seahorse XF Glycolysis Stress Test

The Seahorse XF Glycolysis Stress Test was performed using the Agilent Seahorse XF Glycolysis Stress Test Kit (103020‐100), following the manufacturer's protocol. 1 day before the assay, the Seahorse XFe96 Analyzer (Agilent Technologies) was powered on for equilibration, and the sensor cartridge was hydrated overnight in Seahorse XF Calibrant. XF96 cell culture microplates were coated with poly‐L‐ornithine (PLO, 1:10 dilution) and fibronectin (FN, 1:40 dilution) at 4°C overnight. On the day of the experiment, Seahorse XF Base Medium was supplemented with 2 mM L‐glutamine and adjusted to pH 7.4 to prepare the assay medium. GSCs were resuspended in assay medium and seeded into the coated XF96 plate at 1 × 10^4^ cells/well in 50 µL volume. The plate was incubated in a 37°C non‐CO_2_ incubator for 25–30 min to allow attachment, followed by the addition of 130 µL pre‐warmed assay medium per well and further incubation for 15–25 min. The following compounds were prepared and loaded into the injection ports of the hydrated sensor cartridge: Port A: 20 µL of 100 mM glucose (final concentration: 10 mM), Port B: 22 µL of 10 µM oligomycin (final concentration: 1 µM), Port C: 25 µL of 500 mM 2‐deoxy‐D‐glucose (2‐DG) (final concentration: 50 mM). After calibration, the sensor cartridge was loaded onto the cell plate, and the assay was conducted using the Seahorse XFe96 Analyzer. Following the assay, cells were lysed, and protein concentration was quantified for normalization. Data were analyzed using Wave software (Seahorse Wave Controller 2.6).

### 4.25 Co‐Immunoprecipitation Assay

Co‐IP was performed using the Pierce Co‐IP Kit (88804, Thermo Fisher Scientific) according to the manufacturer's instructions. GSC cells or GBM tissues were lysed in 500 µL of RIPA buffer supplemented with phenylmethanesulfonyl fluoride (PMSF; ST506, Beyotime), followed by centrifugation at 12 000 × g for 15 min at 4°C. The resulting supernatants were incubated with magnetic beads and specific antibodies at 4°C for 12 h. Beads were subsequently washed four times with Co‐IP buffer, and the bound proteins were eluted and subjected to Western blot assay or used for mass spectrometry.

### 4.26 Lactate Dehydrogenase Activity Assay

LDH enzymatic activity was measured using the Lactate Dehydrogenase Activity Assay Kit (MAK066, Sigma‐Aldrich) according to the manufacturer's protocol. To generate the standard curve, 0, 2, 4, 6, 8, and 10 µL of 1.25 mM NADH standard solution were added to a 96‐well plate, and the volume in each well was adjusted to 50 µL with LDH assay buffer. GSCs from different treatment groups were resuspended in LDH assay buffer at a concentration of 1 × 10^6^ cells per 500 µL. The suspension was centrifuged at 10 000 × g for 15 min at 4°C to remove insoluble debris. Then, 10 µL of the supernatant was transferred to a 96‐well plate, and the volume was brought up to 50 µL with assay buffer. Next, 50 µL of reaction mix—consisting of 48 µL LDH assay buffer and 2 µL substrate mix—was added to each well, yielding a total reaction volume of 100 µL. After 2–3 min of pre‐incubation, absorbance at 450 nm (A450) was measured using a microplate reader. The plate was incubated at 37°C, and A450 was recorded every 5 min in the dark. Measurements continued until the absorbance approached or exceeded the maximum value of the NADH standard curve. LDHA activity was calculated by determining the change in absorbance (ΔA450) over time and referencing the standard curve. Enzymatic activity was expressed as the amount of NADH consumed (or generated) per unit time.

### 4.27 In Vitro LDHA Enzymatic Activity Assay

Plasmids encoding LDHA^WT^‐Flag and LDHA^C163A^‐Flag were transfected into 293T cells. 48 h post‐transfection, cells were lysed and LDHA proteins were immunoprecipitated using anti‐Flag affinity beads. Bound proteins were eluted with elution buffer containing 20 mM HEPES, 150 mM NaCl, 1 mM EDTA, and 200 µg/mL 3×Flag peptide (pH 7.5). For the enzyme activity assay, 1 µg of purified LDHA protein was added to a reaction buffer consisting of 0.2 M Tris‐HCl (pH 7.4), 30 mM pyruvate, and 2 mM NADH. The decrease in NADH absorbance was monitored at 340 nm using a microplate reader. For kinetic analysis, reactions were performed in 0.1 M Tris‐HCl buffer, pH 7.4, containing 0.2 mM NADH and increasing concentrations of pyruvate: 0.05, 0.1, 0.2, 0.5, 1, 2, 5 and 10 mM. Reactions were initiated by adding equal amounts of purified LDHA protein. Initial velocities were calculated from the linear decrease in absorbance at 340 nm during the early phase of the reaction. Michaelis‐Menten curves were fitted by nonlinear regression to determine Km and Vmax values.

### 4.28 Molecular Dynamics Simulation

MD simulations were conducted to evaluate the structural stability and dynamic behavior of LDHA in complex with pyruvic acid, both in its wild‐type form and after palmitoylation at Cys163. All simulations were performed using GROMACS v2022.03 (RRID: SCR_014565) with the AMBER99SB‐ILDN force field. The initial structures were obtained from molecular docking results and converted into GROMACS‐compatible formats. The protein–ligand complexes were solvated in a TIP3P water model with a minimum distance of 1.2 nm from the box edge. To mimic physiological conditions, 0.154 M NaCl was added and counterions were used to neutralize the system. Ligand parameters were generated using GAFF via AmberTools22, with RESP charges derived from Gaussian 16 W calculations. Energy minimization was carried out using the steepest descent algorithm. Short‐range electrostatic and van der Waals interactions were truncated at 1.0 nm, and long‐range electrostatics were calculated using the Particle Mesh Ewald (PME) method. All bonds involving hydrogen atoms were constrained using the LINCS algorithm. Temperature and pressure were maintained using V‐rescale and Berendsen barostat, respectively. The systems underwent 100 ps of initial equilibration in an NVT ensemble, followed by 100 ps in an NPT ensemble. Production MD simulations were performed for 200 ns at 300 K and 1 bar. Simulation trajectories were analyzed for root‐mean‐square deviation (RMSD), root‐mean‐square fluctuation (RMSF), B‐factor, and the C‐terminal to N‐terminal distance using built‐in GROMACS tools. Free energy landscapes were generated using g_sham, and binding free energies were estimated using the MM/PBSA method via MMPBSA.py v16.0.

### 4.29 Exosome Isolation

Conditioned media from GSC cells cultures were collected and subjected to sequential centrifugation at 4°C: first at 2000 × g for 30 min and then at 12 000 × g for 45 min to remove cellular debris. The resulting supernatant was ultracentrifuged at 100 000 × g for 70 min at 4°C to pellet exosomes. Exosome pellets were resuspended in PBS and stored at –80°C, avoiding repeated freeze–thaw cycles to preserve integrity.

### 4.30 PBMC Isolation and Induction of Macrophages

Peripheral blood mononuclear cells (PBMCs) were isolated from the peripheral blood of three healthy volunteers as previously described [71]. Briefly, the PBMCs were isolated using standard density gradient centrifugation (2000 rpm, 30 min) from venous blood. Within 15 min of collection, CD14^+^ monocytes were then purified from PBMCs using magnetic CD14 microbeads (130‐050‐201, Miltenyi Biotec) according to the manufacturer's instructions. The isolated monocytes were cultured in RPMI 1640 complete medium supplemented with 100 ng/mL M‐CSF (300‐25‐50UG, Peprotech) for 7 days to induce differentiation into macrophages.

### 4.31 Virtual Screening of Small Molecule Inhibitors Targeting LDHA Cys163

Virtual screening was performed to identify small‐molecule inhibitors targeting the Cys163 site of human LDHA (PDB ID: 5W8J, 1.55 Å). Protein preparation was carried out using the Protein Preparation Wizard in Schrödinger Maestro 12.8 with hydrogen addition and energy minimization (OPLS_2005, RMSD cutoff: 0.30 Å). A receptor grid was generated around Cys163 (box size: 20 Å^3^). Four compound libraries from MedChemExpress were screened: HY‐L028 (∼0.9K), HY‐L901 (50K), HY‐L0055V (50.2K), and HY‐L001V (24.4K). Compounds were processed using LigPrep (RRID: SCR_016746) to generate energy‐minimized 3D structures.

Docking was conducted using the Glide module within the Virtual Screening Workflow. HY‐L028 was screened directly using extra precision (XP) mode. HY‐L901 and HY‐L0055V underwent three‐stage docking (HTVS‐SP‐XP), retaining the top 10% at each stage, while HY‐L001V used a 15% threshold. The top 200 ranked compounds from each library were manually curated based on docking scores and structural features. The top hit, HY‐N0148 (docking score: −9.448), was further analyzed in PyMOL (RRID: SCR_000305). Six key hydrogen bonds were identified with residues VAL270, ARG171, ASN164, and ARG269. Both 2D and 3D interaction diagrams were generated to visualize the binding conformation.

### 4.32 Lactate Quantification Assay

Lactate levels were measured using the Lactate Assay Kit (E‐BC‐K044, Elabscience,) according to the manufacturer's instructions. A lactate standard curve was generated by preparing a series of standard dilutions. GSCs from different treatment groups were resuspended in PBS at a concentration of 1 × 10^6^ cells per 300 µL. After centrifugation at 10 000 × g for 10 min at 4°C, the supernatants were collected and kept on ice for analysis. An aliquot of the supernatant was used to determine protein concentration using the BCA protein assay for normalization. For the assay, 5 µL of each sample or standard solution was added to a 96‐well plate, followed by the addition of 100 µL of enzyme working solution. Subsequently, 20 µL of Reagent III was added, and the plate was incubated at 37°C for 10 min. Then, 180 µL of Reagent IV was added to each well and mixed thoroughly. The absorbance at 530 nm was measured using a microplate reader. Lactate concentrations were calculated based on the standard curve generated from known lactate standards and normalized to protein content where appropriate.

### 4.33 Protein Purification and Surface Plasmon Resonance Analysis

The Homo‐LDHA in pet28a plasmid was obtained from GenePharma and transformed into Escherichia coli BL21 cells (TransGen Biotech, China). Transformed strains were cultured in LB medium containing 0.1 mg/mL kanamycin at 37°C with shaking at 200 rpm. Protein expression was induced with 0.5 mM isopropyl‐β‐D‐thiogalactopyranoside (IPTG; Solarbio, China), and LDHA protein was purified using a Protein Purification Kit (CoWin Bio, Cambridge, MA, USA).

Surface plasmon resonance (SPR) analysis was conducted using the SensiQ Pioneer platform (ForteBio, Fremont, CA, USA). PCH biosensor chips (19‐0129, ForteBio) were first activated with 1 mM NiCl_2_, followed by immobilization of LDHA at a concentration of 50 µg/mL. Binding of the small molecule candidates were assessed, and response units (RU) were recorded to quantify binding activity. Data were normalized to control values and analyzed using Qdat software (ForteBio), and sensorgrams were used to generate binding curves.

### 4.34 Cellular Thermal Shift Assay

Cellular Thermal Shift Assay (CETSA) was performed with minor modifications from previously described protocols [72]. Briefly, cells were trypsinized, washed, and resuspended in PBS. Aliquots (50 µL, approximately 1 × 10^6^ cells) were heated at temperatures ranging from 37°C to 73°C in 4°C increments for 3 min using a thermocycler. Cells were then lysed by three freeze–thaw cycles in liquid nitrogen, followed by incubation on ice for 20 min. Lysates were centrifuged at 20 000 × g for 10 min at 4°C, and the resulting soluble fractions were mixed with 5× SDS loading buffer for subsequent immunoblot analysis.

### 4.35 Single‐Cell RNA Sequencing

To evaluate the role of LDHA palmitoylation inhibition by Rutin in remodeling the immunosuppressive tumor microenvironment of GBM in vivo, Rutin was administered via oral gavage five times per week starting 7 days after GBM cell implantation. scRNA‐seq was conducted following the protocol described in previous studies [73]. Briefly, freshly dissociated single‐cell suspensions were loaded onto the microfluidic chip of the Chip A Single Cell Kit v2.0 (S050100201, MobiDrop), and droplets were generated using the MobiNova‐100 system (A1A40001). Each cell or nucleus was encapsulated in a droplet containing a gel bead linked to millions of oligonucleotides with unique barcodes. After light‐induced cleavage by the MobiNovaSP‐100 (A2A40001), oligonucleotides diffused into the reaction mix, allowing mRNA capture via oligo (dT) sequences on the beads. Barcoded cDNAs were synthesized by reverse transcription, amplified, and used for library construction with the High Throughput Single Cell 3’ RNA‐Seq Kit v2.0 and 3’ Single Index Kit. Libraries were sequenced on the Illumina NovaSeq 6000 platform. Cell Ranger was used to align scRNA‐seq data to the reference genome and quantify individual transcripts for each cell. Seurat (v4.1.0) was then employed to remove low‐quality cells based on user‐defined criteria, followed by data downsampling and clustering. Finally, t‐distributed stochastic neighbor embedding (t‐SNE) was applied for two‐dimensional visualization of cellular distributions.

### 4.36 GBM Organoids Construction

The methods were adapted from a previous study on GBOs [74]. Fresh surgical specimens obtained from GBM patients were mechanically minced and cultured in 6‐well plates containing 2 mL of GBO medium per well. The GBO medium consisted of a 1:1 mixture of DMEM/F12 (A4192001, Gibco) and Neurobasal (21103049, Gibco), supplemented with 1% GlutaMax (35050061, Gibco), 1% non‐essential amino acids (NEAAs), 1% Penicillin‐Streptomycin, 1% N2 supplement (A1370701, Gibco), and 1× B27 supplement (17504044, Gibco). Cultures were maintained on an orbital shaker at 140 rpm in a sterile incubator set to 37°C with 5% CO_2_. Within 2–3 weeks, tissue fragments typically developed into spherical organoid structures suitable for analyses.

### 4.37 Statistics

All statistical analyses were performed using GraphPad Prism version 10 (GraphPad Software, RRID: SCR_002798). Analyses were performed with blinding to group allocation. Data are presented as mean ± standard deviation (SD). For comparisons between two groups, a two‐tailed unpaired Student's *t*‐test was used. For comparisons among multiple groups, one‐way analysis of variance (ANOVA) followed by Tukey's post hoc test was applied. Statistical significance was set at *p* < 0.05, and all statistical tests were two‐tailed. *p < 0.05, **p < 0.01, and ***p < 0.001.

## Author Contributions

G.L., X.Q. and H.X. conceived the project, designed the experiments, and secured funding. Z.J.G. and Z.Y.C. performed the experiments, analyzed the data, and wrote the first draft of the manuscript. R.R.Z., H.X., X.G., and P.Z. contributed to manuscript revision. Q.T.W. and Z.J.G. established and cultured primary glioma cells and organoid models. H.Y.Y., B.Y.L., Y.H.Q., S.L.Z., J.X.Z., J.W.Q., Z.W.P., Z.H., Q.T.W., R.C.L., and H.Z.X. assisted in performing experiments and data analysis. X.G., X.Q., P.Z., G.L., and H.X. supervised the study and contributed to writing and editing the final version of the manuscript. All authors read and approved the final manuscript.

## Conflicts of Interest

The authors declare no conflicts of interest.

## Supporting information




**Supporting File 1**: advs77536‐sup‐0001‐SuppMat.docx.

**Supporting File 2**: advs77536‐sup‐0002‐SuppMat.xlsx.

## Data Availability

The human GBM datasets analyzed in this study were obtained from publicly available repositories, including the TCGA Research Network (http://cancergenome.nih.gov/), CGGA (http://www.cgga.org.cn/), and GEO (GSE16011, GSE108476, and GSE131928; https://www.ncbi.nlm.nih.gov/geo/). The crystal structure of LDHA used in this study is available from the Protein Data Bank (PDB ID: 5W8J). The RNA sequencing and single‐cell RNA sequencing raw data generated in this study have been deposited in GSA‐Human (https://ngdc.cncb.ac.cn/gsa‐human/) under accession numbers HRA017777 and HRA009155, respectively. The proteomics data have been deposited in OMIX (https://ngdc.cncb.ac.cn/omix/) under accession number OMIX016100. Further information and requests for resources and reagents should be directed to the corresponding author, Gang Li (dr.ligang@sdu.edu.cn).

## References

[1] D. N. Louis , A. Perry , P. Wesseling , et al., “The 2021 WHO Classification of Tumors of the Central Nervous System: A Summary,” Neuro‐Oncology 23, no. 8 (2021): 1231–1251, 10.1093/neuonc/noab106. PMC8328013 34185076

[2] Q. T. Ostrom , M. Price , C. Neff , et al., “CBTRUS Statistical Report: Primary Brain and Other Central Nervous System Tumors Diagnosed in the United States in 2015–2019,” Neuro‐Oncology 24, no. 5 (2022): v1–v95, 10.1093/neuonc/noac202. PMC9533228 36196752

[3] A. Shergalis , A. Bankhead , U. Luesakul , N. Muangsin , and N. Neamati , “Current Challenges and Opportunities in Treating Glioblastoma,” Pharmacological Reviews 70, no. 3 (2018): 412–445, 10.1124/pr.117.014944. PMC5907910 29669750

[4] S. Bao , Q. Wu , R. E. McLendon , et al., “Glioma Stem Cells Promote Radioresistance by Preferential Activation of the DNA Damage Response,” Nature 444, no. 7120 (2006): 756–760, 10.1038/nature05236. 17051156

[5] N. Ghosh , D. Chatterjee , and A. Datta , “Tumor Heterogeneity and Resistance in Glioblastoma: The Role of Stem Cells,” Apoptosis 30, no. 7‐8 (2025): 1695–1729, 10.1007/s10495-025-02123-y. 40375039

[6] A. Anand , J. K. Petersen , L. B. Andersen , et al., “Multiomic Profiling of Glioblastoma Metabolic Lesions Reveals Complex Intratumoral Genomic Evolution and Dipeptidase‐1‐driven Vascular Proliferation,” Neuro‐Oncology 27, no. 10 (2025): 2547–2563, 10.1093/neuonc/noaf071. PMC12833548 40319462

[7] J. Zhao , X. Cui , Q. Zhan , et al., “CRISPR‐Cas9 Library Screening Combined With an Exosome‐targeted Delivery System Addresses Tumorigenesis/TMZ Resistance in the Mesenchymal Subtype of Glioblastoma,” Theranostics 14, no. 7 (2024): 2835–2855, 10.7150/thno.92703. PMC11103500 38773970

[8] G. Bourmeau , O. Anezo , J. Raymond , et al., “Proneural–mesenchymal Hybrid Glioblastoma Cells Are Resistant to Therapy and Dependent on Nuclear Import,” Neuro‐Oncology 27, no. 11 (2025): 2876–2893, 10.1093/neuonc/noaf160. PMC12908486 40627673

[9] T. Hara , R. Chanoch‐Myers , N. D. Mathewson , et al., “Interactions between Cancer Cells and Immune Cells Drive Transitions to Mesenchymal‐Like States in Glioblastoma,” Cancer Cell 39, no. 6 (2021): 779–792.e11, 10.1016/j.ccell.2021.05.002. PMC8366750 34087162

[10] J. Bailleul and E. Vlashi , “Glioblastomas: Hijacking Metabolism to Build a Flexible Shield for Therapy Resistance,” Antioxidants & Redox Signaling 39, no. 13‐15 (2023): 957–979, 10.1089/ars.2022.0088. PMC10655009 37022791

[11] C. Lu , T. Kang , J. Zhang , et al., “Combined Targeting of Glioblastoma Stem Cells of Different Cellular States Disrupts Malignant Progression,” Nature Communications 16, no. 1 (2025): 2974, 10.1038/s41467-025-58366-5. PMC11947120 40140646

[12] W. Wang , T. Li , Y. Cheng , et al., “Identification of Hypoxic Macrophages in Glioblastoma with Therapeutic Potential for Vasculature Normalization,” Cancer Cell 42, no. 5 (2024): 815–832.e12, 10.1016/j.ccell.2024.03.013. 38640932

[13] A. Rashidi , L. K. Billingham , A. Zolp , et al., “Myeloid Cell‐Derived Creatine in the Hypoxic Niche Promotes Glioblastoma Growth,” Cell Metabolism 36, no. 1 (2024): 62–77.e8, 10.1016/j.cmet.2023.11.013. PMC10842612 38134929

[14] X. Jin , L. J. Y. Kim , Q. Wu , et al., “Targeting Glioma Stem Cells through Combined BMI1 and EZH2 Inhibition,” Nature Medicine 23, no. 11 (2017): 1352–1361, 10.1038/nm.4415. PMC5679732 29035367

[15] N. Essakhi , A. Bertucci , N. Baeza‐Kallee , et al., “Metabolic Adaptation of Myeloid Cells in the Glioblastoma Microenvironment,” Frontiers in Immunology 15 (2024): 1431112, 10.3389/fimmu.2024.1431112. PMC11700814 39763643

[16] E. Johansson , E. S. Grassi , V. Pantazopoulou , et al., “CD44 Interacts with HIF‐2α to Modulate the Hypoxic Phenotype of Perinecrotic and Perivascular Glioma Cells,” Cell Reports 20, no. 7 (2017): 1641–1653, 10.1016/j.celrep.2017.07.049. 28813675

[17] R. Zhao , Y. Hou , B. Li , et al., “Bioengineered Hybrid Dual‐targeting Nanoparticles Reprogram the Tumour Microenvironment for Deep Glioblastoma Photodynamic Therapy,” Nature Communications 16, no. 1 (2025): 7672, 10.1038/s41467-025-63081-2. PMC12361390 40826003

[18] A. Kricha , N. Bouchmaa , S. Ben Mkaddem , A. Abbaoui , R. Ben Mrid , and R. El Fatimy , “Glioblastoma‐Associated Macrophages: A Key Target in Overcoming Glioblastoma Therapeutic Resistance,” Cytokine & Growth Factor Reviews 80 (2024): 97–108, 10.1016/j.cytogfr.2024.10.009. 39510901

[19] Y. R. Miao , E. B. Rankin , and A. J. Giaccia , “Therapeutic Targeting of the Functionally Elusive TAM Receptor family,” Nature Reviews Drug Discovery 23, no. 3 (2024): 201–217, 10.1038/s41573-023-00846-8. PMC11335090 38092952

[20] A. R. Pombo Antunes , I. Scheyltjens , F. Lodi , et al., “Single‐Cell Profiling of Myeloid Cells in Glioblastoma Across Species and Disease Stage Reveals Macrophage Competition and Specialization,” Nature Neuroscience 24, no. 4 (2021): 595–610, 10.1038/s41593-020-00789-y. 33782623

[21] A. T. Yeo , S. Rawal , B. Delcuze , et al., “Single‐cell RNA Sequencing Reveals Evolution of Immune Landscape during Glioblastoma Progression,” Nature Immunology 23, no. 6 (2022): 971–984, 10.1038/s41590-022-01215-0. PMC9174057 35624211

[22] L. Liu , R. Wang , A. Alifu , et al., “Hypoxia‐Driven M2‐Polarized Macrophages Facilitate the Epithelial‐Mesenchymal Transition of Glioblastoma via Extracellular Vesicles,” Theranostics 14, no. 16 (2024): 6392–6408, 10.7150/thno.95766. PMC11488104 39431006

[23] Z. Qin , X.‐W. Bian , and Y. Shi , “Identification of Hypoxic Macrophages in Glioblastoma: Unveiling Therapeutic Insights From Tumour Microenvironment Analysis,” Clinical and Translational Medicine 14, no. 9 (2024): 70013, 10.1002/ctm2.70013. PMC11412069 39297872

[24] A. Sattiraju , S. Kang , B. Giotti , et al., “Hypoxic Niches Attract and Sequester Tumor‐associated Macrophages and Cytotoxic T Cells and Reprogram Them for Immunosuppression,” Immunity 56, no. 8 (2023): 1825–1843.e6, 10.1016/j.immuni.2023.06.017. PMC10527169 37451265

[25] J. Hu , X. Sa , Y. Yang , et al., “Multi‐Transcriptomics Reveals Niche‐Specific Expression Programs and Endothelial Cells in Glioblastoma,” Journal of Translational Medicine 23, no. 1 (2025): 444, 10.1186/s12967-025-06185-z. PMC11998397 40234880

[26] M. Podhorecka , A. Skladanowski , and P. Bozko , “H2AX Phosphorylation: Its Role in DNA Damage Response and Cancer Therapy,” Journal of Nucleic Acids 2010, no. 1 (2010): 9, 10.4061/2010/920161. PMC2929501 20811597

[27] S. Fatima , X. Hu , R.‐H. Gong , et al., “Palmitic Acid Is an Intracellular Signaling Molecule Involved in Disease Development,” Cellular and Molecular Life Sciences 76, no. 13 (2019): 2547–2557, 10.1007/s00018-019-03092-7. PMC11105207 30968170

[28] M. E. Smith and R. P. Bazinet , “Unraveling Brain Palmitic Acid: Origin, Levels and Metabolic Fate,” Progress in Lipid Research 96 (2024): 101300, 10.1016/j.plipres.2024.101300. 39222711

[29] S.‐H. Kim , R. Ezhilarasan , E. Phillips , et al., “Serine/Threonine Kinase MLK4 Determines Mesenchymal Identity in Glioma Stem Cells in an NF‐κB‐dependent Manner,” Cancer Cell 29, no. 2 (2016): 201–213, 10.1016/j.ccell.2016.01.005. PMC4837946 26859459

[30] Z. Gao , J. Xu , Y. Fan , et al., “ARPC1B Promotes Mesenchymal Phenotype Maintenance and Radiotherapy Resistance by Blocking TRIM21‐Mediated Degradation of IFI16 and HuR in Glioma Stem Cells,” Journal of Experimental & Clinical Cancer Research 41, no. 1 (2022): 323, 10.1186/s13046-022-02526-8. PMC9667586 36380368

[31] M. Qu , X. Zhou , X. Wang , and H. Li , “Lipid‐Induced S‐Palmitoylation as a Vital Regulator of Cell Signaling and Disease Development,” International Journal of Biological Sciences 17, no. 15 (2021): 4223–4237, 10.7150/ijbs.64046. PMC8579454 34803494

[32] L. Zhou , G. Lian , T. Zhou , et al., “Palmitoylation of GPX4 via the Targetable ZDHHC8 Determines Ferroptosis Sensitivity and Antitumor Immunity,” Nat Cancer 6, no. 5 (2025): 768–785. 10.1038/s43018-025-00937-y 40108413

[33] H. Kang , B. Kim , J. Park , H. Youn , and B. Youn , “The Warburg Effect on Radioresistance: Survival Beyond Growth,” Biochimica et Biophysica Acta (BBA)—Reviews on Cancer 1878, no. 6 (2023): 188988, 10.1016/j.bbcan.2023.188988. 37726064

[34] T. Arif , Y. Krelin , I. Nakdimon , et al., “VDAC1 is a Molecular Target in Glioblastoma, with Its Depletion Leading to Reprogrammed Metabolism and Reversed Oncogenic Properties,” Neuro‐Oncology 19, no. 7 (2017): 951–964, 10.1093/neuonc/now297. PMC5570220 28339833

[35] X. Chen , Q. Wu , W. Gong , et al., “GRP75 Triggers White Adipose Tissue Browning to Promote Cancer‐Associated Cachexia,” Signal Transduction and Targeted Therapy 9, no. 1 (2024): 253, 10.1038/s41392-024-01950-w. PMC11427701 39327432

[36] S. Daniele , C. Giacomelli , E. Zappelli , et al., “Lactate Dehydrogenase‐A Inhibition Induces human Glioblastoma Multiforme Stem Cell Differentiation and Death,” Scientific Reports 5, no. 1 (2015): 15556, 10.1038/srep15556. PMC4616042 26494310

[37] L. Yang , S. Li , L. Yu , J. Leng , and N. Li , “Targeting Glycolysis: Exploring a New Frontier in Glioblastoma Therapy,” Frontiers in Immunology 15 (2025): 1522392, 10.3389/fimmu.2024.1522392. PMC11772265 39877360

[38] Z. Zhang , X. Li , F. Yang , et al., “DHHC9‐Mediated GLUT1 S‐palmitoylation Promotes Glioblastoma Glycolysis and Tumorigenesis,” Nature Communications 12, no. 1 (2021): 5872, 10.1038/s41467-021-26180-4. PMC8497546 34620861

[39] L. Zhao , C. Zhang , X. Luo , et al., “CD36 palmitoylation Disrupts Free Fatty Acid Metabolism and Promotes Tissue Inflammation in Non‐Alcoholic Steatohepatitis,” Journal of Hepatology 69, no. 3 (2018): 705–717, 10.1016/j.jhep.2018.04.006. 29705240

[40] Z. Zhang , C. Ren , R. Xiao , et al., “Palmitoylation of TIM‐3 Promotes Immune Exhaustion and Restrains Antitumor Immunity,” Science Immunology 9, no. 101 (2024): adp7302, 10.1126/sciimmunol.adp7302. 39546589

[41] H. Yao , J. Lan , C. Li , et al., “Inhibiting PD‐L1 Palmitoylation Enhances T‐cell Immune Responses Against Tumours,” Nature Biomedical Engineering 3, no. 4 (2019): 306–317, 10.1038/s41551-019-0375-6. 30952982

[42] Q.‐T. Chen , Z.‐Y. Zhang , Q.‐L. Huang , et al., “HK1 From Hepatic Stellate Cell–Derived Extracellular Vesicles Promotes Progression of Hepatocellular Carcinoma,” Nature Metabolism 4, no. 10 (2022): 1306–1321, 10.1038/s42255-022-00642-5. PMC9584821 36192599

[43] B. M. Andersen , C. Faust Akl , M. A. Wheeler , E. A. Chiocca , D. A. Reardon , and F. J. Quintana , “Glial and Myeloid Heterogeneity in the Brain Tumour Microenvironment,” Nature Reviews Cancer 21, no. 12 (2021): 786–802, 10.1038/s41568-021-00397-3. PMC8616823 34584243

[44] C. Neftel , J. Laffy , M. G. Filbin , et al., “An Integrative Model of Cellular States, Plasticity, and Genetics for Glioblastoma,” Cell 178, no. 4 (2019): 835–849.e21, 10.1016/j.cell.2019.06.024. PMC6703186 31327527

[45] P. Pandey , F. Khan , H. A. Qari , and M. Oves , “Rutin (Bioflavonoid) as Cell Signaling Pathway Modulator: Prospects in Treatment and Chemoprevention,” Pharmaceuticals 14, no. 11 (2021): 1069, 10.3390/ph14111069. PMC8621917 34832851

[46] M. Ben Sghaier , A. Pagano , M. Mousslim , Y. Ammari , H. Kovacic , and J. Luis , “Rutin Inhibits Proliferation, Attenuates Superoxide Production and Decreases Adhesion and Migration of human Cancerous Cells,” Biomedicine & Pharmacotherapy 84 (2016): 1972–1978. 10.1016/j.biopha.2016.11.001 27829548

[47] A. Ganeshpurkar and A. K. Saluja , “The Pharmacological Potential of Rutin,” Saudi Pharmaceutical Journal 25, no. 2 (2017): 149–164, 10.1016/j.jsps.2016.04.025. PMC5355559 28344465

[48] A. A. Perk , I. Shatynska‐Mytsyk , Y. C. Gerçek , et al., “Rutin Mediated Targeting of Signaling Machinery in Cancer Cells,” Cancer Cell International 14, no. 1 (2014): 124, 10.1186/s12935-014-0124-6. PMC4260193 25493075

[49] M. Ercelik , C. Tekin , M. Gurbuz , et al., “A New Nano Approach to Prevent Tumor Growth in the Local Treatment of Glioblastoma: Temozolomide and Rutin‐loaded Hybrid Layered Composite Nanofiber,” Asian Journal of Pharmaceutical Sciences 19 (2024): 100971. 10.1016/j.ajps.2024.100971 PMC11617954 39640055

[50] J. Kim , B.‐K. Koo , and J. A. Knoblich , “Human Organoids: Model Systems for human Biology and Medicine,” Nature Reviews Molecular Cell Biology 21, no. 10 (2020): 571–584, 10.1038/s41580-020-0259-3. PMC7339799 32636524

[51] G. Zhu , Z. Sun , Y. Liu , et al., “Rational Design and Organoid‐Based Evaluation of a Cocktail CAR‐Γδ T Cell Therapy for Heterogeneous Glioblastoma,” Advanced Science 12, no. 19 (2025): 2501772, 10.1002/advs.202501772. PMC12097020 40112194

[52] H. K. Brar , J. Jose , Z. Wu , and M. Sharma , “Tyrosine Kinase Inhibitors for Glioblastoma Multiforme: Challenges and Opportunities for Drug Delivery,” Pharmaceutics 15, no. 1 (2022): 59, 10.3390/pharmaceutics15010059. PMC9863099 36678688

[53] M. D. Wood , G. F. Reis , D. E. Reuss , and J. J. Phillips , “Protein Analysis of Glioblastoma Primary and Posttreatment Pairs Suggests a Mesenchymal Shift at Recurrence,” Journal of Neuropathology & Experimental Neurology 75, no. 10 (2016): 925–935, 10.1093/jnen/nlw068. PMC6281078 27539476

[54] G. Pascual , D. Domínguez , M. Elosúa‐Bayes , et al., “Dietary Palmitic Acid Promotes a Prometastatic Memory via Schwann Cells,” Nature 599, no. 7885 (2021): 485–490, 10.1038/s41586-021-04075-0. 34759321

[55] S. Deng , J. Wang , F. Zou , et al., “Palmitic Acid Accumulation Activates Fibroblasts and Promotes Matrix Stiffness in Colorectal Cancer,” Cancer Research 85, no. 10 (2025): 1784–1802. 10.1158/0008-5472.CAN-24-2892 PMC12079102 39992719

[56] Y. Fukata and M. Fukata , “Protein Palmitoylation in Neuronal Development and Synaptic Plasticity,” Nature Reviews Neuroscience 11, no. 3 (2010): 161–175, 10.1038/nrn2788. 20168314

[57] R. Kang , J. Wan , P. Arstikaitis , et al., “Neural Palmitoyl‐Proteomics Reveals Dynamic Synaptic Palmitoylation,” Nature 456, no. 7224 (2008): 904–909, 10.1038/nature07605. PMC2610860 19092927

[58] G. S. Brigidi and S. X. Bamji , “Detection of Protein Palmitoylation in Cultured Hippocampal Neurons by Immunoprecipitation and Acyl‐biotin Exchange (ABE),” Journal of Visualized Experiments 72 (2013): 50031, 10.3791/50031. PMC3605617 23438969

[59] S. Yang , M. Li , G. Lian , Y. Wu , J. Cui , and L. Wang , “ABHD8 Antagonizes Inflammation by Facilitating Chaperone‐Mediated Autophagy‐Mediated Degradation of NLRP3,” Autophagy 21, no. 2 (2025): 338–351, 10.1080/15548627.2024.2395158. PMC11759624 39225180

[60] L. Wang and J. Cui , “Palmitoylation Promotes Chaperone‐mediated Autophagic Degradation of NLRP3 to Modulate Inflammation,” Autophagy 19, no. 10 (2023): 2821–2823, 10.1080/15548627.2023.2187957. PMC10472840 36927399

[61] L. Wang , J. Cai , X. Zhao , et al., “Palmitoylation Prevents Sustained Inflammation by Limiting NLRP3 Inflammasome Activation Through Chaperone‐mediated Autophagy,” Molecular Cell 83, no. 2 (2023): 281–297.e10, 10.1016/j.molcel.2022.12.002. 36586411

[62] L. Wang , M. Li , G. Lian , et al., “Palmitoylation Acts as a Checkpoint for MAVS Aggregation to Promote Antiviral Innate Immune Responses,” Journal of Clinical Investigation 134, no. 23 (2024): 177924, 10.1172/JCI177924. PMC11601910 39621307

[63] F. Khan , Y. Lin , H. Ali , et al., “Lactate Dehydrogenase A Regulates Tumor‐Macrophage Symbiosis to Promote Glioblastoma Progression,” Nature Communications 15, no. 1 (2024): 1987, 10.1038/s41467-024-46193-z. PMC10914854 38443336

[64] L. Bu , Z. Zhang , J. Chen , et al., “High‐Fat Diet Promotes Liver Tumorigenesis via Palmitoylation and Activation of AKT,” Gut 73, no. 7 (2024): 1156–1168, 10.1136/gutjnl-2023-330826. 38191266

[65] Q. Yue , B. He , Z. Guo , N. Zhang , M. Zhang , and Y. Zhang , “Exploration of Ultrasound‐Enhanced Transdermal Delivery Efficiency and Anti‐Inflammatory Effect of Rutin,” Pharmaceuticals 18, no. 4 (2025): 464, 10.3390/ph18040464. PMC12030127 40283901

[66] T. Andonova , Y. Muhovski , S. Naimov , et al., “Potentillae Argenteae Herba‐Antioxidant and DNA‐Protective Activities, and Microscopic Characters,” Antioxidants (Basel) 14, no. 4 (2025): 487. 10.3390/antiox14040487 PMC12023972 40298827

[67] L. Wu , H. Lin , S. Li , et al., “Macrophage Iron Dyshomeostasis Promotes Aging‐Related Renal Fibrosis,” Aging Cell 23, no. 11 (2024): 14275, 10.1111/acel.14275. PMC11561705 39016438

[68] J.‐C. Huang , X.‐K. Pan , S.‐C. Li , et al., “Polyphenol Extracts From Rambutan Peel Promote Longevity via the Attenuation of the Toll/Imd Pathway,” Food & Function 16, no. 7 (2025): 2793–2807, 10.1039/D4FO06353H. 40094275

[69] H. Huang , J. Shi , W. Chen , and L. Liu , “Rutin Suppresses the Malignant Biological Behavior of Gastric Cancer Cells Through the Wnt/β‐catenin Pathway,” Discover Oncology 15, no. 1 (2024): 407, 10.1007/s12672-024-01281-w. PMC11374940 39231903

[70] M. Ercelik , C. Tekin , F. N. Parin , et al., “Co‐Loading of Temozolomide With Oleuropein or Rutin Into Polylactic Acid Core‐Shell Nanofiber Webs Inhibit Glioblastoma Cell by Controlled Release,” International Journal of Biological Macromolecules 253 (2023): 126722, 10.1016/j.ijbiomac.2023.126722. 37673167

[71] Z. Zhang , J. Xu , Z. Chen , et al., “Transfer of MicroRNA via Macrophage‐Derived Extracellular Vesicles Promotes Proneural‐to‐Mesenchymal Transition in Glioma Stem Cells,” Cancer Immunology Research 8, no. 7 (2020): 966–981, 10.1158/2326-6066.CIR-19-0759. 32350000

[72] J. Li , J. Liu , Z. Zhou , et al., “Tumor‐Specific GPX4 Degradation Enhances Ferroptosis‐Initiated Antitumor Immune Response in Mouse Models of Pancreatic Cancer,” Science Translational Medicine 15, no. 720 (2023): adg3049, 10.1126/scitranslmed.adg3049. 37910602

[73] K. Zhang , Y. Wang , S. Chen , et al., “TREM2hi Resident Macrophages Protect the Septic Heart by Maintaining Cardiomyocyte Homeostasis,” Nature Metabolism 5, no. 1 (2023): 129–146, 10.1038/s42255-022-00715-5. PMC9886554 36635449

[74] F. Jacob , R. D. Salinas , D. Y. Zhang , et al., “A Patient‐Derived Glioblastoma Organoid Model and Biobank Recapitulates Inter‐ and Intra‐Tumoral Heterogeneity,” Cell 180, no. 1 (2020): 188–204.e22, 10.1016/j.cell.2019.11.036. PMC7556703 31883794

