# Supplementary material for: Targeting LDHA Palmitoylation Sensitizes GBM to Chemoradiotherapy via Suppressing Palmitic Acid Induced Glycolytic Reprogramming
Source: Adv Sci (Weinh). 2026 Sep 28:e77536. Online ahead of print. doi: 10.1002/advs.77536 (PMC13618168; doi:10.1002/advs.77536)
Supplement: Supplementary file 1 [file ADVS-9999-e77536-s002.docx]

Supplementary Materials for

**Targeting LDHA palmitoylation sensitizes GBM to chemoradiotherapy via suppressing palmitic acid induced glycolytic reprogramming**

Zijie Gao *et al*

*Correspondence: xqianmedres@njmu.edu.cn (X.Q.), dr.ligang@sdu.edu.cn (G.L.), xuehao@sdu.edu.cn (H.X.)

**SUPPLEMENTARY FIGURES AND FIGURE LEGENDS**

**Fig. S1. Integrated single-cell analysis identifies the pivotal role of mesenchymal GBM cells in tumor recurrence and therapeutic resistance.**

(**A**) t-SNE plot of single-cell RNA-seq data showing the major cell types within GBM specimens, including endothelial cells (EC), fibroblasts (FB), malignant cells, oligodendrocytes (OLIG), T cells, and tumor-associated macrophages (TAMs). (**B**) Heatmap of hallmark gene sets enriched across distinct GBM subpopulations. (**C**) Proportion of major malignant subtypes (AC, OPC, MES1/2, NPC) under newly diagnosed (ND) versus recurrent disease (RD) conditions (left) and across tumor core versus periphery (right). (**D**) Schematic workflow of the experimental design: GBM resection specimens were cultured, followed by orthotopic transplantation and treatment with standard chemoradiotherapy, and then subjected to transcriptomic and proteomic profiling. (**E**) Flow cytometric analysis of apoptosis cells percent in control (Ctrl) and standard chemoradiotherapy (SCRT) group with or without treatment, n=3. (**F** and **G**) Representative images and quantification of comet assay showing reduced DNA damage (tail moment), n=40. Scale bar, 50 µm. Data shown are mean ± SD Error bars, *p < 0.05, **p < 0.01, ***p < 0.001.

**Fig. S2. Mesenchymal GBM cells are characterized by enhanced fatty acid uptake and transport activity.**

(**A**) Heatmap of single-sample GSEA (ssGSEA) scores for metabolic pathway signatures in Ctrl and SCRT groups. (**B**) GSEA based on scRNA-seq data showing that fatty acid import and metabolic processes are specifically enriched in MES1-like GBM cells. (**C**) Dot plot of single-cell transcriptome data showing fatty acid transport (FAT) activity across different glioma cell clusters. (**D**) Quantification of FAT activity across the four GBM cell subtypes (AC, MES, NPC, OPC). (**E**) Targeted metabolomic profiling reveals enrichment of specific fatty acids in GBM tissues, n=8. Data shown are mean ± SD Error bars, *p < 0.05, **p < 0.01, ***p < 0.001.

**Fig. S3. SLC27A3 drives enhanced palmitate uptake in mesenchymal GSCs and correlates with poor prognosis.**

(**A**) Representative images of BODIPY FL C16-labeled PN and MES subtype GSCs demonstrating FAT efficiency. Scale bar, 10 μm. (**B**) Flow cytometric analysis of BODIPY FL C16 uptake at different time points (1, 10, 30 min) in representative MES (GSC 267, 20) and PN (GSC 8-11, 11) GSC lines. (**C**) Quantification of relative FAT activity over time, showing enhanced uptake kinetics in MES GSCs compared to PN GSCs, n=3. (**D**) Density plot of spatial transcriptomics data highlighting enrichment of the long-chain fatty acid import pathway. (**E**) Representative images of BODIPY FL C16 staining demonstrating markedly impaired fatty acid uptake following SLC27A3 knockdown in GSC 267 and GSC 20. Scale bar, 5 μm. (**F**) Relative expression of SLC27A3 in PN vs MES GBM subtypes from TCGA and Rembrandt datasets, showing significantly higher expression in MES subtype. (**G**) Kaplan–Meier survival analysis indicating that high SLC27A3 expression is associated with worse prognosis in GBM patients. (**H**) Immunoblot validation of SLC27A3 protein expression in MES GSC lines (GSC 267, GSC 20, GSC 28) vs PN GSC lines (GSC 8-11, GSC 11) and normal neural progenitor cells (NPC). (**I**) Heatmaps showing differential mRNA expression of SLC27 family genes in Ctrl and SCRT group tumor cells. Data shown are mean ± SD Error bars, *p < 0.05, **p < 0.01, ***p < 0.001.

**Fig. S4. PA promotes MES subtype maintenance and chemoradiotherapy resistance of GSCs.**

(**A**) Immunoblot analysis showing PA dose-dependent upregulation of MES markers (CD44, LYN, Vimentin) in GSC 20 and GSC 267. (**B**) Representative images of the co-culture model comprising tumor spheroids and normal rat brain organoids. Tumor cells pretreated with PA exhibited enhanced invasive capacity, n=3. Scale bar, 200 μm. (**C**) Immunoblot showing that PA induces MES markers in GSCs, which is blocked by SLC27A3 knockdown. (**D**) Flow cytometric analysis of apoptosis cells percent in GSC 20 and GSC 267. Fatty acid deficiency (Dialysis medium, DM) enhances chemoradiotherapy-induced apoptosis, which is partially reversed by PA supplementation, n=3. (**E**) Representative comet assay (n=40; Scale bar, 50 μm,) and γ-H2AX IF (n=3; Scale bar, 5 μm) staining images and quantification showing that PA treatment significantly reduces DNA damage in GSC 267 post-chemoradiotherapy. (**F**) Flow cytometry quantification of apoptotic cells percent to assess the impact of PA on therapeutic sensitivity in GSC 267, n=3. (**G**) In vivo bioluminescence imaging of intracranial GSC 267 xenograft-bearing mice fed with control diet or high-palmitate diet (HPD), with or without chemoradiotherapy on day 7. Quantification on day 7 indicates no significant difference in total photon flux between the different groups, n=5. Data shown are mean ± SD Error bars, *p < 0.05, **p < 0.01, ***p < 0.001.

**Fig. S5. PA promotes therapeutic resistance primarily by driving glycolytic reprogramming through palmitoylation.**

(**A**) Quantification of Annexin V+ apoptotic cells in GSC 267 across different treatment groups, n=3. (**B**) Representative images of comet assay (upper, Scale bar, 50 μm) and γ-H2AX IF staining (lower, Scale bar, 5 μm) in GSC 267 cells after indicated treatments. (**C**) Quantification of DNA damage by comet tail moment (left, n=40) and γ-H2AX foci per nucleus (right, n=3) in GSC 267. PA reduced chemoradiotherapy-induced DNA damage, an effect reversed by 2-BP. (**D**) Enrichment analysis of potential palmitoylated proteins, highlighting key role in glycolysis regulation. (**E**) Seahorse analysis of extracellular acidification rate (ECAR) in GSC lines. Quantification of glycolysis, glycolytic capacity, and glycolytic reserve shows that MES GSCs (GSC 267, GSC 20 and GSC 28) exhibit higher glycolytic capacity and reserve than PN GSCs (GSC 8-11 and GSC 11). (**F**) Seahorse ECAR analysis of glycolytic function in GSC 267 treated with PA and/or 2-BP. (**G**) Quantification of lactate LA production in GSC 267 under indicated treatments. (**H**) Seahorse analysis of ECAR in SCRT and control tumor cells with or without 2-BP pretreatment. SCRT tumor cells display elevated glycolysis, which is suppressed by 2-BP treatment. Data shown are mean ± SD Error bars, *p < 0.05, **p < 0.01, ***p < 0.001.

**Fig. S6. ZDHHC12 is positively correlated with glycolysis and poor prognosis in GBM, especially in the MES subtype.**

**(A)** Correlation heatmap showing the association between glycolysis signature scores and the expression of ZDHHC family members in the TCGA GBM dataset. Among them, ZDHHC12 exhibits the strongest positive correlation with glycolytic activity. **(B)** Representative scatter plots showing the positive correlation between glycolysis activity score and expression of ZDHHC12, ZDHHC3, and ZDHHC5. **(C)** Boxplots comparing expression of ZDHHC family members between MES and PN GBM subtypes in the TCGA GBM dataset. ZDHHC12 expression is significantly upregulated in MES subtype. **(D)** Forest plot of hazard ratios (HRs) from univariable Cox regression analysis for ZDHHC family genes in TCGA GBM. (**E**) Heatmap visualizing the RNA expression profiles of ZDHHC family members in Ctrl and SCRT groups. **(F** and **G)** Proteomic analysis of SCRT and control GBM cells, showing significantly upregulated ZDHHC12 and ZDHHC13 expression in the SCRT group. **(H** and **I)** Flow cytometric analysis of Annexin V+ apoptotic cells upon ZDHHC12 or ZDHHC13 knockdown (**H**, SCRT tumor cells) or overexpression (**I**, control tumor cells), n=3. **(J)** Kaplan-Meier survival analyses across four independent GBM cohorts show that high ZDHHC12 expression is associated with worse overall survival. **(K)** Expression analysis of ZDHHC12 across GBM subtypes in four public datasets. ZDHHC12 is consistently upregulated in MES compared to PN subtype GBM. Data shown are mean ± SD Error bars, *p < 0.05, **p < 0.01, ***p < 0.001.

**Fig. S7. ZDHHC12 mediates PA-induced glycolytic reprogramming and chemoradiotherapy resistance in GSCs.**

**(A)** IF staining analysis of γ-H2AX (red) foci in GSC 267 cells post chemoradiotherapy after knockdown of ZDHHC12. DAPI (blue), ZDHHC12 (green). Quantification shows increased γ-H2AX foci per cell in knockdown groups, indicating enhanced DNA damage, n=3. Scale bar, 10 μm. **(B)** Representative γ-H2AX staining and quantification of GSC 267 cells treated with PA in NC and ZDHHC12-knockdown conditions, n=3. Scale bar, 10 μm. (**C**) Seahorse ECAR analysis in GSC 267 under indicated conditions. ZDHHC12 knockdown suppresses PA-induced glycolysis, glycolytic capacity, and glycolytic reserve. (**D**) The measurement of lactate levels for indicated groups, n=3. Data shown are mean ± SD Error bars, *p < 0.05, **p < 0.01, ***p < 0.001.

**Fig. S8. ZDHHC12 knockdown enhances mitochondrial respiration and increases ROS accumulation in GSCs**

**(A** and **B)** Seahorse XF analysis of mitochondrial respiration parameters in GSC 20 and GSC 267 cells after knockdown of ZDHHC12. OCR (oxygen consumption rate) curves were measured following sequential injection of oligomycin, FCCP, and rotenone. Quantification of basal respiration, maximal respiration, proton leak, and ATP production are shown (right panels). **(C)** Representative images of ROS levels in GSC 20 and GSC 267 cells after chemoradiotherapy, detected by ROS-specific dye (green). Quantification of mean fluorescence intensity (MFI) is shown in the right bar graphs, n=3. Scale bar, 50 μm. (**D**) Schematic model showing that ZDHHC12 knockdown shifts PA-driven glycolysis toward mitochondrial respiration, leading to elevated ROS accumulation in GSCs. Data shown are mean ± SD Error bars, *p < 0.05, **p < 0.01, ***p < 0.001.

**Fig. S9. ZDHHC12 promotes chemoradiotherapy resistance of GBM in vivo.**

**(A)** In vivo bioluminescence imaging of orthotopic GSC 267 tumors in nude mice on the seventh day after surgery. Quantification of total photon flux on day 7 indicated no significant difference in initial tumor burden between groups, n=5. (**B**) Representative images of H&E staining (Scale bar, 1 mm) and IHC (Scale bar, 50 μm) for ZDHHC12 and γ-H2AX in brain tumor sections from each group. Data are presented as mean ± sd. *P < 0.05, **P < 0.01, ***P < 0.001 by Student’s t-test or one-way ANOVA as appropriate.

**Fig. S10. ZDHHC12 catalyzes LDHA palmitoylation at Cys163.**

**(A)** Representative immunoblots of ABE assays detecting palmitoylated VDAC1, PKM2, SLC25A5 and LDHA in GSC20 and GSC267 cells. **(B)** Co-immunoprecipitation (Co-IP) analysis showing the interaction between ZDHHC12 and LDHA in GSC 267. **(C)** ABE assay detecting LDHA palmitoylation. Knockdown of ZDHHC12 inhibited PA induced LDHA palmitoylation. (**D**) Immunoblot analysis showing hypoxia treatment enhance the interaction of ZDHHC12 and LDHA. (**E**) Swiss-Palm analysis revealed five conserved potential palmitoylation sites in LDHA, among which Cys163 was functionally validated as the critical modification site. (**F**) Schematic overview of the design strategy for LDHA mutants. (**G**) Immunoblot analysis of LDHA palmitoylation by ABE assay in HEK293T cells transfected with plasmids encoding WT LDHA-Flag or its indicated mutants. (**H**) Immunoblot analysis demonstrating that LDHA^WT^, but not the palmitoylation-deficient mutant LDHA^C163A^, restored MES markers expression. (**I**) Co-IP analysis showing that Cys163 mutation does not disrupt the ZDHHC12–LDHA interaction. (**J**) Representative proximity ligation assay (PLA) images and quantification showing the interaction between ZDHHC12 and Flag-LDHA^WT^ or LDHA^C163A^, n=3. Scale bar, 20 μm. Data are presented as mean ± sd. *P < 0.05, **P < 0.01, ***P < 0.001 by Student’s t-test or one-way ANOVA as appropriate.

**Fig. S11. LDHA Cys163 palmitoylation modulates its structural conformation, enzymatic activity, and substrate-binding dynamics.**

(**A**) LDHA protein levels in GSC 20 and GSC 267 treated with PA, 2-BP, or both. (**B**) IF staining of LDHA in GSCs under the indicated treatments revealed no significant changes in its subcellular localization. Scale bar, 10 μm. (**C**) In vitro enzyme kinetic analysis of LDHA^WT^ and LDHA^C163A^ with or without palmitoyl-CoA treatment. Palmitoyl-CoA increased the Vmax and decreased the Km of LDHA^WT^, but had little effect on LDHA^C163A^, n=3. (**D**-**H**) Molecular dynamics simulations of WT LDHA and palmitoylated LDHAC163, including: (**D**) binding conformation, (**E**) binding distance maps, (**F**) residue-level binding energy contribution, (**G**) Gibbs energy landscapes, and (**H**) total binding free energy comparison. Data shown are mean ± SD Error bars, *p < 0.05, **p < 0.01, ***p < 0.001.

**Fig. S12. Association of myeloid-derived macrophages enrichment with tumor recurrence and hypoxia in GBM.**

(**A**) Transmission electron microscopy (TEM) images of exosomes isolated from GSC 267 cells treated with DMSO or PA. Scale bar, 100 nm. (**B**) Nanoparticle tracking analysis (NTA) showing the size distribution of exosomes. (**C**) Rescue experiments with Flag-LDHA^WT^ or palmitoylation-deficient LDHA^C163A^ mutant demonstrating that LDHA palmitoylation is required for its incorporation into EVs. (**D**) t-SNE plot of single-cell transcriptomes distinguishing myeloid-derived macrophages (MDMs) and microglia (MG). (**E** and **F**) Density plots of ND versus RD samples and inner versus periphery samples. (**G**) Density map showing the spatial distribution of MDMs. (**H**) Dot plot showing upregulated genes in MDMs compared with MG. (**I**) GSEA analysis identifying key pathways enriched in MDMs.

**Fig. S13. Hypoxia-associated MDMs exhibit distinct pathway enrichment in recurrent GBM.**

(**A**) Heatmap of hallmark pathway activities in MDMs across tumor core versus periphery, highlighting enrichment of glycolysis, hypoxia, EMT, TNFA–NFκB signaling, and angiogenesis in MDM subpopulations. (**B**) Heatmap comparing MDMs from ND and RD GBM tissues, showing enhanced activation of hypoxia, glycolysis, inflammatory response, and DNA repair pathways in recurrent tumors.

**Fig. S14. ZDHHC12 knockdown in GSCs impairs exosome-mediated induction of immunosuppressive macrophages and suppresses tumor growth.**

(**A**) Proportions of MDM subpopulations in ND versus RD GBM and in tumor core versus periphery. (**B**) Violin plots of immunosuppressive markers across macrophage subpopulations, highlighting higher expression in RD-associated MDMs. (**C**) Experimental workflow design: NC or ZDHHC12 knockdown GSCs were treated with PBS or PA, followed by exosome isolation and co-culture with THP1- or PBMC-derived macrophages to assess immunosuppressive phenotypes. (**D**) Flow cytometry of THP1-derived macrophages showing that exosomes from PA-treated GSCs significantly increase CD163⁺ and CD206⁺ macrophages, whereas ZDHHC12 knockdown attenuates this effect. (**E**) Flow cytometry analysis showing that EVs from LDHA^WT^-rescued GSCs restored the expression of CD163 and CD206, whereas EVs from LDHA^MUT^-expressing cells failed to do so. (**F**) qPCR analysis of inflammatory cytokine expression in macrophages treated with exosomes from indicated GSCs, n=3. (**G**) ELISA quantification of secreted IL-1β in PBMC-derived macrophages after exosome treatment, n=3. (**H**) Immunoblotting showing increased p-p65 and SLC27A3 expression in GSC20 and GSC267 cells following IL-1β treatment at 0, 10, and 20 ng/mL. (**I**) NF-κB inhibition by BMS-345541 attenuated IL-1β induced p-p65 and SLC27A3 upregulation. (**J**) Representative images of BODIPY FL C16-labeled GSCs demonstrating FAT efficiency. Scale bar, 10 μm. (**K**) Flow cytometric analysis of BODIPY FL C16 uptake at indicated groups, n=3. (**L**) In vivo bioluminescence imaging and quantification of total photon flux showing that knockdown of ZDHHC12 reduced tumor burden, n=5. (**M**) Survival analysis showing that knockdown of ZDHHC12 significantly prolonged survival, n=5. Data shown are mean ± SD Error bars, *p < 0.05, **p < 0.01, ***p < 0.001.

**Fig. S15. Rutin suppresses LDHA-mediated glycolysis and enhances therapeutic-induced DNA damage in GSCs.**

(**A**) SPR screening of candidate small molecules binding to LDHA. Rutin exhibited the highest binding affinity. (**B**) Immunoblot analysis of cleaved Caspase3 in GSC 267 cells treated with increasing concentrations of Rutin (0-200 μM) in combination with chemoradiotherapy, indicating enhanced apoptosis. (**C**) Cellu1lar thermal shift assay (CETSA) showing increased thermal stability of LDHA in the presence of Rutin, suggesting direct binding. (**D**) Immunoblot analysis showing Rutin significantly inhibit the expression of MES markers expression in GSC 20 and GSC 267. (**E**) Co-IP analysis showing the interaction between ZDHHC12 and LDHA in GSCs treated with vehicle or Rutin at 50 and 100 μM. (**F**) Representative PLA images and quantification showing that Rutin treatment did not significantly affect ZDHHC12-LDHA proximity. Red puncta indicate PLA-positive signals; nuclei were stained with DAPI, n=3. Scale bar, 10 μm. (**G**) ABE assay showing that Rutin reduced ZDHHC12-mediated palmitoylation of LDHA^WT^, whereas LDHA^C163A^ showed minimal palmitoylation signal. (**H**) Seahorse analysis of ECAR in GSC 267 cells. Right: quantification of glycolysis, glycolytic capacity, and glycolytic reserve. (**I**) Intracellular lactate concentration measured in GSC 267 cells under indicated treatments. (**J**) Comet assay showing DNA damage in GSC 267 cells with or without chemoradiotherapy and/or Rutin treatment. Right: quantification of tail moment, n=40. Scale bar, 50 μm. (**K**) IF staining of γH2AX (red) in GSC 267 cells under indicated conditions. Right: quantification of γH2AX foci, n=3. Scale bar, 5 μm. Data shown are mean ± SD Error bars, *p < 0.05, **p < 0.01, ***p < 0.001.

**Fig. S16. Rutin treatment reduces immunosuppressive macrophage infiltration and alters the tumor microenvironment.**

(**A**) Representative in vivo bioluminescent images of GL261-bearing mice treated with vehicle control (Ctrl) or Rutin at day 7 and day 28, n=5. (**B**) Quantification of total photon flux at day 7 shows no significant difference between groups (ns, not significant), n=5. **(C)** IF staining of showing decreased infiltration of Cd163 and Cd206 positive macrophages following Rutin administration. Scale bar, 20 μm. (**D**) Gating strategy for flow cytometric analysis of tumor-infiltrating immune cells. Cells were gated on size and granularity (SSC-H vs FSC-H), singlets (FSC-H vs FSC-A), and live Cd45⁺ cells. Cd11b⁺ myeloid cells were further gated for Cd163⁺ and CD206⁺ macrophage subsets. (**E**) Quantification of Cd163^+^ and Cd206⁺ macrophages in Ctrl and Rutin-treated tumors, n=3. Rutin significantly reduced the proportion of both Cd163⁺ and Cd206⁺ macrophages. Data are presented as mean ± sd. *P < 0.05, **P < 0.01, ***P < 0.001 by Student’s t-test or one-way ANOVA as appropriate.
